# Side Chain Effects on the Lipophilicity-antimicrobial-toxicity Correlation of Greener 4-Alkoxy/Amino-7-Chloroquinolines

**DOI:** 10.2174/0109298673372039250614231629

**Published:** 2025-10-08

**Authors:** Gabriela F. Fiss, Everton P. Silva, Maria F. S. Madruga, Abraão P. Sousa, Helivaldo D. S. Souza, Rádamis B. Castor, Maria H. Nascimento, Krystyna G. Lira, Petrônio F. Athayde-Filho

**Affiliations:** 1 Laboratório de Pesquisa em Bioenergia e Síntese Orgânica (LPBS), Department of Chemistry, Federal University of Paraíba, João Pessoa, Brazil;; 2 Department of Molecular Biology, Federal University of Paraíba, João Pessoa, Brazil

**Keywords:** Antibiotics, *Artemia salina*, 7-chloroquinolines, drug research, fungicides, green chemistry

## Abstract

**Background:**

More robust 4-substituted 7-chloroquinolines have been investigated for their diverse properties. However, there is still no systematic study that correlates the effects of the side chain at the 4-position of chloroquine and hydroxychloroquine derivatives with their lipophilicity, antimicrobial and toxicity properties.

**Objective:**

To this end, a cleaner and facile approach was planned to obtain nineteen 4- substituted 7-chloroquinolines, whose influence of the substituent group and side chain extension at the 4-position on their properties was studied.

**Methods:**

4-Alkoxy/amino-7-chloroquinolines were prepared by a nucleophilic aromatic substitution (S_N_Ar) reaction between 4,7-dichloroquinoline and alcohols/amines, evaluated for their *in silico* ADMET test, *in vitro* antimicrobial activity against Gram-(+) and Gram-(−) bacteria, and *Candida albicans* fungus, and *in vitro* toxicity on *Artemia salina* larvae.

**Results:**

4-Alkoxy/amino-7-chloroquinolines were obtained in yields ranging from 81 to 100%. The best results showed antimicrobial activity against *Pseudomonas aeruginosa* for 4-amino-7-chloroquinolines **6-8**, with halos greater than 20 mm, and against *C. albicans* for 4-amino-7-chloroquinolines **1-3**, with halos close to 30 mm. A correspondence between Minnow toxicity prediction and *in vitro* toxicity on *A. salina* larvae was observed, where compounds **3** and **14**, with R = Pent, were both predicted to have high acute toxicity (log LC_50_ < -0.3) and classified as highly toxic (LC_50_ < 100 µg mL^-1^). It seems that increased lipophilicity in the side chain is harmful to *A. salina* larvae.

**Conclusion:**

Considering the results for compounds **1-3** and **6-8** with greater activity against *C. albicans* and *P. aeruginosa*, respectively, especially for 4-amino-7-chloroquinolines **6** and **7**, which are slightly toxic on *A. salina* larvae (LC_50_ 500-1000 µg mL^-1^), their antimicrobial studies deserve to be continued by the determination of Minimum Inhibitory Concentration (MIC) values.

## 1 INTRODUCTION

Nitrogen is the most common heteroatom in heterocyclic compounds, which have been used in pharmaceuticals such as antibiotics [1] and antitumor [2]. Considering the most recent commercial data on nitrogenous heterocycles, they were the 32^nd^ most traded product in the world in 2022 [3]. Nitrogenous heterocycles include quinoline and its derivatives. Among the quinoline derivatives, some clinical drugs deserve to be highlighted, such as Bedaquiline, antitubercular, Chloroquine and Hydroxychloroquine, antimalarial, Ciprofloxacin, antibiotic, Pitavastatin, cholesterol reducer, among others [4-8].

Structure-activity relationships in aminoquinolines have suggested that the alkylamino and chloro groups at positions 4 and 7, respectively, are essential for antiplasmodial activity [9]. In another study, the alkyl side chain between three and six carbons in 4-amino-7-chloroquinolines was shown to be more selective and active against malaria [10]. Furthermore, the scientific community has registered several synthetic [11, 12] and supramolecular [13, 14] research on 4-amino-7-chloroquinolines, as well as their antimicrobial properties [15-17].

The variation in the number of carbons in the alkyl chain, *i.e.*, variation in lipophilicity, has been used to modulate microbial resistance mechanisms [18]. It is known that the partition of organic compounds in an octanol:water system is a suitable mimic of the membrane:water interface. Therefore, lipophilicity, which has a direct impact on oral absorption, permeability and toxicity, can be predicted through this partition coefficient, where the measurement of the concentrations of a substance in both phases results in log P_O/W_ (log C_O_/C_W_), whose ideal range is between values ​​1 and 3 [19].

The pharmacokinetic phase, which encompasses the Absorption, Distribution, Metabolism and Excretion (ADME) processes, can be drastically affected by the variation in the physicochemical properties of a drug. One of the main physicochemical properties of a micromolecule capable of altering its therapeutic profile is the partition coefficient (P), which expresses the relationship between its hydro/liposolubility profiles. In this context, Lipinski and collaborators [20, 21] proposed a set of predictive parameters, all ideally multiples of five and, therefore, known as the Rule of Five [22]. In addition, Veber and collaborators contributed to the prediction of additional properties for the oral bioavailability profile of drug candidates [23].

According to Lipinski, these rules have been questioned due to the fact that they were proposed at a time when most drugs had been discovered through phenotypic approaches, *i.e.*, based on the observable effects of these drugs on organisms. Currently, the most common approach to drug development is based on the therapeutic target, considering a specific mechanism of action, *in silico* fragment screening and *in vitro* tests [24]. Prediction of pharmacokinetic properties is a guide but should not be limiting. Promising compounds that violate one or more parameters must be tested appropriately, considering the whole. Ultimately, the numbers speak and direct us towards more assertive paths.

More robust 4-substituted 7-chloroquinolines have been investigated regarding the influence of the alkyl side chain on their properties [10]. On the other hand, derivatives more similar to chloroquine and hydroxychloroquine [25, 26] have been used as synthetic intermediates, but there is still no systematic study that correlates the effects of the substituent group and alkyl chain at the 4-position on the lipophilicity, antimicrobial and toxicity properties.

4,7-Dichloroquinoline is a very favorable substrate to undergo a nucleophilic aromatic substitution (S_N_Ar) reaction through the 4-position. Thus, 4-substituted 7-chloroquinolines have been prepared from the S_N_Ar reaction of 4,7-dichloroquinoline with different nucleophiles, generally under adverse conditions of high temperature (T > 100°C), long reaction time (t > 12 h), additional solvent or purification step [11]. Therefore, there is still no standardized cleaning method that covers a more extensive series.

Our research group has been engaged in developing processes and/or chemical products that prevent environmental pollution [27-29]. Now, with the aim of studying the influence of the side chain on lipophilicity, antimicrobial and toxicity properties, a cleaner and facile approach was developed to obtain nineteen 4-alkoxy/amino-7-chloroquinolines, which were evaluated for their *in silico* ADMET test and *in vitro* antimicrobial activity against Gram-(+) and Gram-(−) bacteria, and *Candida albicans* fungus. Furthermore, *in vitro* toxicity on *Artemia salina* larvae for the most active compounds was required.

## 2 MATERIALS AND METHODS

### 2.1 Chemistry

All common reagents were purchased from commercial suppliers and used without further purification. Melting points were measured using QUIMIS equipment, model Q340S23 (Diadema, Brazil). ^1^H and ^13^C Nuclear Magnetic Resonance (NMR) spectra were performed at Laboratório Multiusuário de Caracterização e Análise (LMCA-UFPB), which were acquired on Bruker Ascend (Coventry, United Kingdom) and Bruker (Billerica, MA. USA) spectrometers at 400 and 500 MHz, respectively, for ^1^H, at 298 K, using 5 mm tubes. Infrared (IR) spectra were performed at Laboratório de Síntese Orgânica Medicinal (LASOM-UFPB), which were acquired on a Shimadzu spectrometer (Kyoto, Japan), model IRPrestige-21, using KBr pellets. High-Resolution Mass Spectrometry (HRMS) analyses were performed at LMCA-UFPB, using a Shimadzu HPLC (Kyoto, Japan) coupled to a Bruker MicroTOF II (Billerica, MA, USA) with an electrospray ion (ESI) source, and reported as *m/z* (relative intensity) for the molecular ion [M+H]^+^. Acquisition Parameters: Ion Polarity Positive, Capillary 4500 V, End Plate Offset -500 V, Nebulizer 4.0 Bar, Dry Heater 200°C, Dry Gas 8.0 L min^-1^, Divert Valve Waste.

#### 2.1.1 General Procedure for the Synthesis of 4-amino-7-chloroquinolines (1-12)

A mixture of amine (8 mL) and 4,7-dichloroquinoline (10 mmol, 1.98 g) was maintained under magnetic stirring at 80°C for 4 h (for methylamine/propylamine, at 50°C for 12 h), which was monitored by thin layer chromatography (TLC) in ethyl acetate (AcOEt). Afterwards, the reactional mixture was cooled to room temperature, added to a beaker containing ice water (20 mL) and left in the freezer until freezing point. Then, the precipitate formed was filtered and washed with excess ice water. 4-Amino-7-chloroquinolines (**1-12**) were obtained in yields ranging from 81 to 100%.

7-Chloro-*N*-methylquinolin-4-amine (**1**) in 81% yield (8.1 mmol, 1.56 g); white solid; m.p.: 249-250°C ([30]. 245-246°C); ^13^C NMR (126 MHz, DMSO-*d_6_*): *δ* = 152.0, 150.9, 148.8, 133.3, 127.5, 124.1, 123.8, 117.4, 98.4 (9 × C_Ar_), 29.2 (CH_3_) ppm ([12]. (100 MHz, CD_3_OD): *δ* = 153.8, 152.6, 149.6, 136.4, 127.7, 126.1, 124.3, 118.9, 99.4 (9 × C_Ar_), 29.9 (CH_3_) ppm).

7-Chloro-*N*-propylquinolin-4-amine (**2**) in 94% yield (9.4 mmol, 2.07 g); white solid; m.p.: 146-148°C ([31]. 148-148.5°C); ^13^C NMR (126 MHz, CDCl_3_): *δ* = 152.1, 149.9, 149.2, 134.9, 128.8, 125.3, 121.0, 117.2, 99.1 (9 × C_Ar_), 45.1 (CH_2_), 22.2 (CH_2_), 11.7 (CH_3_) ppm ([12]. (125 MHz, CD_3_OD): *δ* = 152.8, 152.3, 149.7, 136.3, 128.8, 125.9, 124.3, 118.8, 99.6 (9 × C_Ar_), 45.8 (CH_2_), 22.6 (CH_2_), 11.9 (CH_3_) ppm).

7-Chloro-*N*-pentylquinolin-4-amine (**3**) in 88% yield (8.8 mmol, 2.18 g); beige solid; m.p.: 119-121°C ([32]. 119-121°C); ^1^H NMR (500 MHz, DMSO-*d_6_*): *δ* = 8.41 (m, 2H, 2 x H_Ar_), 8.29 (bs, 1H, NH), 7.86 (d, *J* = 2.1 Hz, 1H, H_Ar_), 7.53 (dd, *J* = 9.0, 2.3 Hz, 1H, H_Ar_), 6.60 (d, *J* = 6.3 Hz, 1H, H_Ar_), 3.34 (q, *J* = 7.3 Hz, 2H, CH_2_), 1.64 (p, *J* = 7.4 Hz, 2H, CH_2_), 1.33 (m, 4H, 2 x CH_2_), 0.86 (t, *J* = 7.1 Hz, 3H, CH_3_) ppm ([32]. (300 MHz, CDCl_3_): *δ* = 8.53 (d, *J* = 5.4 Hz, 1H, H_Ar_), 7.96 (s, 1H, H_Ar_), 7.64 (d, *J* = 8.9 Hz, 1H, H_Ar_), 7.35 (d, *J* = 8.9 Hz, 1H, H_Ar_), 6.41 (d, *J* = 5.3 Hz, 1H, H_Ar_), 5.0 (bs, 1H, NH), 3.25 (m, 2H, CH_2_), 1.76 (m, 2H, CH_2_), 1.43 (m, 4H, 2 x CH_2_), 0.95 (t, *J* = 7.2 Hz, 3H, CH_3_) ppm); ^13^C NMR (126 MHz, CDCl_3_): *δ* = 152.5, 147.9, 144.4, 135.5, 125.3, 124.9, 123.7, 116.6, 98.6 (9 × C_Ar_), 42.8 (CH_2_), 28.8 (CH_2_), 27.4 (CH_2_), 22.0 (CH_2_), 14.0 (CH_3_) ppm (not found) .

7-Chloro-*N*-(2-(dimethylamino)ethyl)quinolin-4- amine (**4**) in 94% yield (9.4 mmol, 2.34 g); beige solid; m.p.: 124-126°C ([8]. 122-124°C); ^13^C NMR (126 MHz, CDCl_3_): *δ* = 152.0, 150.0, 149.1, 134.9, 128.6, 125.3, 121.6, 117.4, 99.2 (9 × C_Ar_), 57.0 (CH_2_), 45.1 (CH_3_)_2_, 39.9 (CH_2_) ppm ([33]. (101 MHz, DMSO-*d_6_*): *δ* = 151.9, 150.0, 149.1, 133.4, 127.5, 124.1, 124.0, 117.4, 98.7 (9 × C_Ar_), 56.9 (CH_2_), 45.3 (CH_3_)_2_, 40.5 (CH_2_) ppm).

7-Chloro-*N*-(2-(diethylamino)ethyl)quinolin-4- amine (**5**) in 93% yield (9.3 mmol, 2.58 g); beige solid; m.p.: 106-108°C ([34]. 102-103°C); ^13^C NMR (126 MHz, CDCl_3_): *δ* = 152.1, 150.0, 149.1, 134.9, 128.7, 125.3, 121.3, 117.5, 99.3 (9 × C_Ar_), 50.7 (CH_2_), 46.6 (CH_2_)_2_, 39.8 (CH_2_), 12.1 (CH_3_)_2_ ppm ([34]. (50 MHz, CDCl_3_): *δ* = 152.0, 149.8, 149.0, 134.6, 128.5, 125.1, 121.1, 117.3, 99.2 (9 × C_Ar_), 50.4 (CH_2_), 46.3 (CH_2_)_2_, 39.6 (CH_2_), 11.9 (CH_3_)_2_ ppm.

*N*-(2-Aminoethyl)-7-chloroquinolin-4-amine (**6**) in 93% (9.3 mmol, 2.06 g) yield; white solid; m.p.: 149-151°C ([35]. 140-142°C); ^13^C NMR (126 MHz, DMSO-*d_6_*): *δ* = 151.9, 150.3, 149.0, 133.3, 127.4, 124.1, 123.9, 117.4, 98.7 (9 × C_Ar_), 46.0 (CH_2_), 40.0 (CH_2_) ppm ([35]. (100 MHz, CD_3_OD): *δ* = 152.9, 152.5, 149.7, 136.4, 127.6, 126.1, 124.4, 118.9, 99.8 (9 × C_Ar_), 46.3 (CH_2_), 40.9 (CH_2_) ppm).

*N*-(3-Aminopropyl)-7-chloroquinolin-4-amine (**7**) in 94% yield (9.4 mmol, 2.21 g); white solid; m.p.: 96-98°C ([36]. 96-98°C); ^13^C NMR (101 MHz, DMSO-*d_6_*, 80°C): *δ* = 151.5, 149.9, 148.8, 133.0, 127.2, 123.5, 123.5, 117.2, 98.3 (9 × C_Ar_), 40.4 (CH_2_), 40.4 (CH_2_), 31.1 (CH_2_) ppm ([9]. (75 MHz, CDCl_3_): *δ* = 151.5, 150.0, 148.6, 133.9, 127.6, 124.2, 122.0, 117.1, 97.8 (9 × C_Ar_), 42.8 (CH_2_), 40.8 (CH_2_), 29.5 (CH_2_) ppm).

*N*-(4-Aminobutyl)-7-chloroquinolin-4-amine (**8**) in 99% yield (9.9 mmol, 2.47 g); white solid; m.p.: 119-121°C ([36]. 122-124°C); ^13^C NMR (101 MHz, DMSO-*d_6_*, 80°C): *δ* = 151.5, 149.9, 148.9, 133.0, 127.2, 123.6, 123.5, 117.2, 98.3 (9 × C_Ar_), 42.2 (CH_2_), 41.0 (CH_2_), 30.5 (CH_2_), 25.1 (CH_2_) ppm ([9]. (75 MHz, CDCl_3_): *δ* = 151.9, 150.0, 149.0, 134.5, 128.3, 124.8, 121.6, 117.3, 98.6 (9 × C_Ar_), 43.0 (CH_2_), 41.4 (CH_2_), 30.6 (CH_2_) 25.9 (CH_2_) ppm).

*N*-(6-Aminohexyl)-7-chloroquinolin-4-amine (**9**) in 100% yield (10 mmol, 2.77 g); white solid; m.p.: 130-132°C ([36]. 133-134°C); ^13^C NMR (126 MHz, DMSO-*d_6_*): *δ* = 151.9, 150.1, 149.1, 133.3, 127.4, 124.1, 123.9, 117.4, 98.5 (9 × C_Ar_), 42.3 (CH_2_), 41.4 (CH_2_), 32.9 (CH_2_), 27.8 (CH_2_), 26.5 (CH_2_), 26.2 (CH_2_) ppm ([9]. (75 MHz, CD_3_OD): *δ* = 152.3, 149.6, 136.2, 127.5, 125.8, 124.3, 120.2, 118.7, 99.5 (9 × C_Ar_), 43.9 (CH_2_), 42.3 (CH_2_), 33.5 (CH_2_) 29.3 (CH_2_), 28.0 (CH_2_), 27.7 (CH_2_) ppm).

2-(7-Chloroquinolin-4-ylamino)ethanol (**10**) in 89% yield (8.9 mmol, 1.98 g); white solid; m.p.: 225-227°C ([37]. 203-205°C); ^13^C NMR (126 MHz, DMSO-*d_6_*): *δ* = 151.9, 150.2, 149.1, 133.4, 127.4, 124.0, 124.0, 117.4, 98.7 (9 × C_Ar_), 58.7 (CH_2_), 45.1 (CH_2_) ppm ([37]. (126 MHz, DMSO-*d_6_*): *δ* = 152.3, 150.7, 149.5, 133.8, 127.9, 124.4, 124.4, 117.9, 99.1 (9 × C_Ar_), 59.2 (CH_2_), 45.6 (CH_2_) ppm).

3-(7-Chloroquinolin-4-ylamino)propan-1-ol (**11**) in 97% yield (9.7 mmol, 2.29 g); white solid; m.p.: 155-157°C ([37]. 149-150°C); ^13^C NMR (126 MHz, DMSO-*d_6_*): *δ* = 151.9, 150.1, 149.0, 133.4, 127.4, 124.0, 124.0, 117.4, 98.6 (9 × C_Ar_), 58.6 (CH_2_), 39.6 (CH_2_), 31.0 (CH_2_) ppm ([37]. (126 MHz, DMSO-*d_6_*): *δ* = 152.3, 150.6, 149.5, 133.8, 127.8, 124.5, 124.4, 117.9, 99.0 (9 × C_Ar_), 59.0 (CH_2_), 40.0 (CH_2_), 31.4 (CH_2_) ppm).

2-(2-(7-chloroquinolin-4-ylamino)ethylamino)ethanol (**12**) in 91% yield (9.1 mmol, 2.41 g); white solid; m.p.: 142-144°C ([38]. 139-139.5°C); ^13^C NMR (101 MHz, DMSO-*d_6_*): *δ* = 151.9, 150.1, 149.0, 133.3, 127.4, 124.0, 124.0, 117.4, 98.7 (9 × C_Ar_), 60.5 (CH_2_), 51.5 (CH_2_), 47.3 (CH_2_), 42.7 (CH_2_) ppm (not found).

#### 2.1.2 General Procedure for the Synthesis of 4-alkoxy-7-chloroquinolines (13-19)

A mixture of potassium *tert*-butoxide (10 mmol, 1.12 g), alcohol (6 mL) and 4,7-dichloroquinoline (10 mmol, 1.98 g) was maintained under magnetic stirring at 100°C for 8 h, which was monitored by TLC in AcOEt. Afterwards, the reactional mixture was cooled to room temperature, added to a beaker containing ice water (20 mL) and left in the freezer until freezing point. Then, the precipitate formed was filtered and washed with excess ice water. 4-Alkoxy-7-chloroquinolines (**13-19**) were obtained in yields ranging from 81 to 99%. Full NMR, IR and HRMS spectra for 4-alkoxy-7-chloroquinolines (**14**, **18** and **19**) are available in Figs. **(S1-12)**.

7-Chloro-4-propoxyquinoline (**13**) in 81% yield (8.1 mmol, 1.79 g); white solid; m.p.: 70-72°C ([39]. 77-79°C); ^13^C NMR (126 MHz, CDCl_3_): *δ* = 161.8, 152.6, 149.8, 135.7, 127.9, 126.5, 123.6, 120.0, 101.0 (9 × C_Ar_), 70.2 (CH_2_), 22.3 (CH_2_), 10.6 (CH_3_) ppm (not found).

7-Chloro-4-(pentyloxy)quinoline (**14**, C_14_H_16_ClNO) in 88% yield (8.8 mmol, 2.19 g); *R*_f_: 0.42 (AcOEt); beige solid; m.p.: 39-41°C; ^1^H NMR (500 MHz, CDCl_3_): *δ* = 8.69 (d, *J* = 5.2 Hz, 1H, H_Ar_), 8.13 (d, *J* = 8.9 Hz, 1H, H_Ar_), 8.00 (d, *J* = 2.1 Hz, 1H, H_Ar_), 7.42 (dd, *J* = 8.9, 2.0 Hz, 1H, H_Ar_), 6.69 (d, *J* = 5.3 Hz, 1H, H_Ar_), 4.16 (t, *J* = 6.4 Hz, 2H, CH_2_), 1.93 (m, 2H, CH_2_), 1.52 (m, 2H, CH_2_), 1.43 (m, 2H, CH_2_), 0.96 (t, *J* = 7.2 Hz, 3H, CH_3_) ppm; ^13^C NMR (126 MHz, CDCl_3_): *δ* = 161.8, 152.6, 149.8, 135.7, 127.9, 126.5, 123.6, 120.0, 101.0 (9 × C_Ar_), 68.8 (CH_2_), 28.6 (CH_2_), 28.3 (CH_2_), 22.5 (CH_2_), 14.1 (CH_3_) ppm; IR (KBr): *ν* = 3035 (H_Ar_), 2954, 2927, 2862 (H_alkanic_), 1612 (C=N), 1581, 1566, 1500, 1469, 1427 (C=C), 1307, 1195, 1157, 1118, 1068 (C-O), 840, 813, 748 (Ar), 644 (C-Cl) cm^-1^; HRMS (ESI): calcd for C_14_H_17_ClNO ([M+H]^+^) 250.0993, found 250.0993.

2-(7-Chloroquinolin-4-yloxy)ethanol (**15**) in 86% yield (8.6 mmol, 1.92 g); white solid; m.p.: 122-124°C ([37]. 119-120°C); ^13^C NMR (126 MHz, DMSO-*d_6_*): *δ* = 161.1, 153.1, 149.2, 134.4, 127.2, 126.1, 124.2, 119.5, 102.1 (9 × C_Ar_), 70.6 (CH_2_), 59.3 (CH_2_) ppm ([37]. (126 MHz, DMSO-*d_6_*): *δ* = 161.4, 152.2, 149.4, 135.8, 127.6, 126.5, 123.2, 119.6, 101.5 (9 × C_Ar_), 70.0 (CH_2_), 60.8 (CH_2_) ppm).

3-(7-Chloroquinolin-4-yloxy)propan-1-ol (**16**) in 86% yield (8.6 mmol, 2.04 g); white solid; m.p.: 119-121°C ([37]. 112-115°C); ^13^C NMR (126 MHz, DMSO-*d_6_*): *δ* = 160.9, 153.1, 149.1, 134.4, 127.2, 126.2, 123.8, 119.4, 102.0 (9 × C_Ar_), 65.7 (CH_2_), 57.1 (CH_2_), 31.7 (CH_2_) ppm ([37]. (126 MHz, DMSO-*d_6_*): *δ* = 161.4, 152.1, 149.1, 135.7, 127.3, 126.3, 123.2, 119.5, 100.7 (9 × C_Ar_), 65.1 (CH_2_), 58.4 (CH_2_), 31.7 (CH_2_) ppm).

4-(7-Chloroquinolin-4-yloxy)butan-1-ol (**17**) in 99% yield (9.9 mmol, 2.49 g); white solid; m.p.: 150-152°C (not found); ^13^C NMR (101 MHz, DMSO-*d_6_*) *δ* = 160.8, 153.1, 149.1, 134.3, 127.2, 126.2, 123.7, 119.4, 102.1 (9 × C_Ar_), 68.5 (CH_2_), 60.3 (CH_2_), 28.9 (CH_2_), 25.1 (CH_2_) ppm ([9]. (75 MHz, CDCl_3_): *δ* = 161.6, 152.5, 149.5, 135.5, 127.8, 126.3, 123.3, 119.8, 100.9 (9 × C_Ar_), 68.5 (CH_2_), 62.5 (CH_2_), 29.4 (CH_2_), 25.8 (CH_2_) ppm).

2-(2-(7-Chloroquinolin-4-yloxy)ethoxy)ethanol (**18**, C_13_H_14_ClNO_3_) in 82% yield (8.2 mmol, 2.19 g); *R*_f_: 0.42 (AcOEt); white solid; m.p.: 82-84°C; ^1^H NMR (400 MHz, CDCl_3_): *δ* = 8.68 (d, *J* = 5.3 Hz, 1H, H_Ar_), 8.08 (d, *J* = 8.9 Hz, 1H, H_Ar_), 8.01 (dd, *J* = 2.1, 0.4 Hz, 1H, H_Ar_), 7.39 (dd, *J* = 8.9, 2.1 Hz, 1H, H_Ar_), 6.64 (d, *J* = 5.3 Hz, 1H, H_Ar_), 4.30 (m, 2H, CH_2_), 4.00 (m, 2H, CH_2_), 3.82 (m, 2H, CH_2_), 3.73 (m, 2H, CH_2_) ppm; ^13^C NMR (101 MHz, CDCl_3_): *δ* = 161.5, 152.4, 149.6, 135.9, 127.8, 126.6, 123.5, 119.7, 100.9 (9 × C_Ar_), 73.0 (CH_2_), 69.2 (CH_2_), 68.1 (CH_2_), 61.8 (CH_2_) ppm; IR (KBr): *ν* = 3228 (O-H), 3055 (H_Ar_), 2947, 2924, 2866 (H_alkanic_), 1616 (C=N), 1581, 1500, 1431 (C=C), 1311, 1199, 1161, 1126, 1064 (C-O), 837, 821, 767, 748 (Ar), 644 (C-Cl); HRMS (ESI): calcd for C_13_H_15_ClNO_3_ ([M+H]^+^) 268.0735, found 268.0742.

2-(2-(7-Chloroquinolin-4-yloxy)ethylthio)ethanol (**19**, C_13_H_14_ClNO_2_S) in 85% yield (8.5 mmol, 2.41 g); *R*_f_: 0.65 (AcOEt); white solid; m.p.: 107-109°C; ^1^H NMR (500 MHz, CDCl_3_): *δ* = 8.73 (d, *J* = 5.2 Hz, 1H, H_Ar_), 8.13 (d, *J* = 9.0 Hz, 1H, H_Ar_), 8.03 (d, *J* = 2.1 Hz, 1H, H_Ar_), 7.46 (dd, *J* = 8.9, 2.1 Hz, 1H, H_Ar_), 6.73 (d, *J* = 5.3 Hz, 1H, H_Ar_), 4.38 (t, *J* = 6.6 Hz, 2H, CH_2_), 3.83 (t, *J* = 5.9 Hz, 2H, CH_2_), 3.10 (t, *J* = 6.6 Hz, 2H, CH_2_), 2.88 (t, *J* = 5.9 Hz, 2H, CH_2_) ppm; ^13^C NMR (126 MHz, CDCl_3_): *δ* = 161.2, 152.5, 149.9, 136.0, 128.0, 126.9, 123.4, 119.8, 101.1 (9 × C_Ar_), 68.3 (CH_2_), 61.0 (CH_2_), 36.0 (CH_2_), 30.7 (CH_2_) ppm; IR (KBr): *ν* = 3182 (O-H), 2939, 2850 (H_alkanic_), 1612 (C=N), 1573, 1500, 1427 (C=C), 1311, 1276, 1199, 1157, 1122, 1076, 1049, 1018 (C-O/S), 848, 817, 748 (Ar), 644 (C- Cl); HRMS (ESI): calcd for C_13_H_15_ClNO_2_S ([M+H]^+^) 284.0507, found 284.0516.

### 2.2 *In Silico* ADMET Test

The prediction of pharmacokinetic properties, Absorption, Distribution, Metabolism and Excretion (ADME) for 4-alkoxy/amino-7-chloroquinolines **1-19** was carried out through the open-access electronic site SwissADME (http://www.swissadme.ch/). Lipophilicity was deduced from consensus log P (average of all five predictions). The toxicity prediction for 4-alkoxy/amino-7-chloroquinolines **1-19** was carried out through the open-access electronic site pkCSM (https://biosig.lab.uq.edu.au/pkcsm/prediction).

### 2.3 *In Vitro* Antimicrobial Evaluation

4-Alkoxy/amino-7-chloroquinolines (**1-19**) were evaluated for their *in vitro* antimicrobial activity against six microorganisms from American Type Culture Collection (ATCC), Coleção de Culturas Tropical (CCT) and NEWPROV (NEWP), including Gram-(+) (*Bacillus cereus* CCT 0198, *Enterococcus faecalis* NEWP 0012 and *Staphylococcus aureus* ATCC 25923) and Gram-(−) bacteria (*Escherichia coli* NEWP 0039 and *Pseudomonas aeruginosa* NEWP 0027), and *Candida albicans* (NEWP 0031) fungus, according to Antibiotic Susceptibility Testing by a Standardized Single Disk Method [40] and Clinical and Laboratory Standards Institute (CLSI), document M27-A2 [41]. Methanolic solutions were prepared at a concentration of 2 mg mL^-1^. Antibiotic controls used against bacteria included Amoxicillin/Clavulanic acid, Ciprofloxacin, Penicillin G, Tetracycline and Vancomycin, and Fluconazole was used as antifungal control. All experiments were performed in triplicate.

### 2.4 *In Vitro* Toxicity Evaluation

*In vitro* toxicity evaluation of 4-amino-7-chloroquinolines (**1-3** and **6-8**) and 4-alkoxy-7-chloroquinolines (**13-14**) on *Artemia salina* larvae was performed according to an adapted method reported by Sousa *et al.* [42], with some adjustments. In a rectangular aquarium of 3 L capacity and using 20 W light at a height of 20 cm from the water surface, 1 g of *A. salina* cysts was placed in 1 L of saline solution (30 g L^-1^, pH ~ 8) and aerated at 27°C for 48 h. The toxicity was evaluated at concentrations of 125, 250 and 375 µg mL^-1^. To this end, stock solutions were prepared at a concentration of 12.5 mg mL^-1^ for each compound, from which volumes of 50, 100 and 150 µL were captured, added to tubes and, when necessary, completed with DMSO q.s.p. 150 µL (3%). Next, 100 µL (2%) of Tween 80 was added and completed with saline solution q.s.p. 5 mL. Then, ten healthy *A. salina* nauplii were placed inside each tube. After 24 h, the number of alive nauplii was counted. Concentration series (125, 250 and 375 µg mL^-1^) of potassium dichromate solution was used as a positive control. Two negative controls were prepared, one containing only saline solution, and another containing saline solution with 3% DMSO and 2% Tween 80. All experiments were performed in triplicate. Data are expressed in terms of mortality percentage *versus* concentration sample. The percentage of lethality on *A. salina* (%LAS) of the samples was determined by the formula %LAS = [(number of dead nauplii in the test − number of dead nauplii in the negative control) / number of alive nauplii in the negative control] × 100 [43]. Average percentages of alive *A. salina* nauplii in different concentrations were calculated using GraphPad Prism 5.0 (San Diego, CA, USA). Means were significantly different when *p* ≤ 0.0002*** using One-way analysis of variance and Bonferroni’s Multiple Comparison Test (Fig. **S13**). Median lethal concentration (LC_50_) was estimated using linear regression equation (Table **S1**).

## 3 RESULTS

### 3.1 Chemistry

**Scheme 1:**
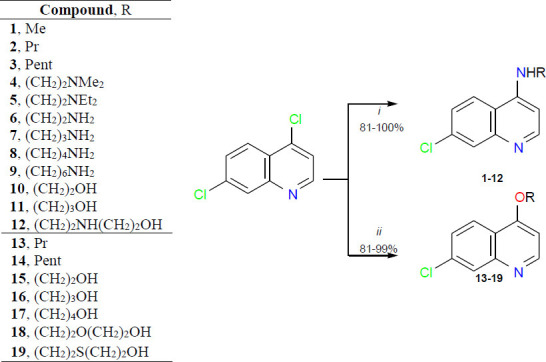
Reagents and reactional conditions to obtain 4-amino-7-chloroquinolines (**1-12**): (*i*) amine, 80°C, 4 h (R = Me/Pr, at 50°C for 12 h); Reagents and reactional conditions to obtain 4-alkoxy-7-chloroquinolines (**13-19**): (*ii*) alcohol, *t*-BuOK, 100°C, 8 h.

Standardized approaches were developed to synthesize twelve 4-amino-7-chloroquinolines (**1-12**) and seven 4-alkoxy-7-chloroquinolines (**13-19**) from an S_N_Ar reaction of 4,7-dichloroquinoline with amines and alcohols, respectively (Scheme **1**).

The novel compounds (**14**, **18** and **19**) were characterized by spectroscopic techniques of IR, and ^1^H and ^13^C NMR. Furthermore, high resolution mass analyses were required.

### 3.2 *In Silico* ADMET Test

Based on Lipinski's Rule of Five and parameters established by Veber, the ADMET prediction [44] for 4-alkoxy/amino-7-chloroquinolines (**1-19**) was generated from SwissADME [45] pkCSM [46] programs, which is available in Table **1**.

### 3.3 *In Vitro* Antimicrobial Evaluation

*In vitro* antimicrobial activity of 4-alkoxy/amino-7-chloroquinolines (**1-19**) was evaluated against three Gram-(+) bacteria, two Gram-(−) bacteria and one yeast fungus. Amoxicillin/Clavulanic acid, Ciprofloxacin, Penicillin G, Tetracycline and Vancomycin were used as antibiotic controls, and Fluconazole as an antifungal control. Zone of inhibition (ZOI) values are available in Table **2**.

### 3.4 *In Vitro* Toxicity Evaluation

Considering the compounds that showed the most relevant antimicrobial activity, an environmental toxicity evaluation was required. Then, *in vitro* toxicity of 4-amino-7-chloroquinolines (**1-3** and **6-8**) and 4-alkoxy-7-chloroquinolines (**13-14**) on *Artemia salina* larvae was evaluated. A saline solution, and a saline solution containing 3% DMSO and 2% Tween 80, in which the number of dead nauplii was equal to 0.33, were used as negative controls. A potassium dichromate solution was used as a positive control, in which all nauplii died. Average percentages of alive nauplii in different concentrations and median lethal concentration (LC_50_) values are provided in Tables **3** and **4**, respectively.

LC_50_ values were compared with those recommended in the literature [47], where LC_50_ values ​​lower than 100 µg mL^-1^ are classified as highly toxic, between 100 and 500 µg mL^-1^ are moderately toxic, between 500 and 1000 µg mL^-1^ are slightly toxic, and above 1000 µg mL^-1^ are non-toxic.

## 4 DISCUSSION

According to Scheme **1**, amines, including diamines and amino alcohols, and alcohols, including diols, were used as reagents and solvents under mild conditions, with reaction temperature equal to or less than 100°C and time no longer than 12 h. 4-Amino-7-chloroquinolines (**1-12**) and 4-alkoxy-7-chloroquinolines (**13-19**) were obtained from precipitation in water in high yields of 81-100% and 81-99%, respectively. Additional steps such as liquid-liquid extraction and/or chromatographic column were not necessary.

In general, 4-alkoxy/amino-7-chloroquinolines have been prepared through the S_Ar_N reaction between 4,7-dichloroquinoline and alcohols/amines (Table **5**) with the addition of solvent [9, 30, 32, 34, 37, 38] or another reagent [9, 37-39], in high temperature [33, 36, 38], long reaction time [9, 30, 32, 33, 37, 39] or additional purification step [9, 32-34, 36-38].

By definition, the key aspect in green chemistry is prevention and reduction [48, 49], such as decreasing the number of reaction steps [50], reactions with green solvents [51, 52] or, preferably, solvent-free [53, 54], safe reaction conditions and short reaction times [55], as well as easy workup processes and high yields. In this work, in compliance with the 1st principle of green chemistry, prevention was achieved, *i.e.*, no additional purification step was required. Moreover, according to principle 5, we avoided the use of solvent, making synthetic processes more economical and environmentally friendly.

In ^1^H NMR spectra for novel compounds **14**, **18** and **19**, when compared to the precursor 4,7-dichloroquinoline, the main signals that confirm the obtaining of the products are those attributed to methylene hydrogens, which appear in the ranges of 4.16-1.43, 4.30-3.73 and 4.38-2.88 ppm, respectively. In addition, the permanence of a set of five signals in the aromatic region was observed. For compound **14**, a triplet attributed to methyl hydrogens was observed at 0.96 ppm.

In ^13^C NMR spectra, methylene carbons were observed in the ranges of 68.8-22.5, 73.0-61.8 and 68.3-30.7 ppm for compounds **14**, **18** and **19**, respectively. For compound **14**, a signal attributed to methyl carbon was observed at 14.1 ppm. Finally, nine aromatic carbons were observed in the range of 161.8 to 100.9 ppm. Comparing carbon shifts at position-4 between products and precursor, they appeared at lower fields (161.8-161.2 ppm) due to the higher electronegativity of oxygen compared to chlorine.

Considering IR spectra, the most significant bands were the emergence of alkyl bands at 2954-2850 cm^-1^, as well as symmetric/asymmetric stretching and in- plane/out-of-plane angular deformations relative to C-O/S at 1311-1006 cm^-1^. Moreover, for compounds **18** and **19**, typical broad bands were observed at 3228 and 3182 cm^-1^, respectively, relative to O-H.

According to Table **1**, all compounds have molecular weights between 192.64 and 283.77 Da, hydrogen bond donors between 0 and 3, and hydrogen bond acceptors between 1 and 4. The partition coefficient log P, a measure of lipo/hydrosolubility ideally no greater than 5, presented values between 1.98 and 4.01. The range between 1 and 3 is optimal for good intestinal absorption, due to the adequate balance between hidrosolubility and permeability. Compounds in the range between 3 and 5 have high permeability, but oral absorption is reduced due to decreased hidrosolubility [18].

In the same way as for Lipinski's Rule of Five, all compounds are in accordance with Veber parameters, with topological polar surface area between 22.12 and 67.65 Å^2^, and number of rotatable bonds between 1 and 7.

Considering the toxicity prediction, the skin sensitization parameter is expressed as yes or no [46]. All compounds are predicted not to cause skin sensitization, indicating a possible topical use. Hepatotoxicity is a parameter that predicts whether a molecule has the potential to cause liver problems, and its result is also expressed as yes or no [46]. For the 4-amino-7-chloroquinoline series, compounds are predicted to be hepatotoxic when, in the side chain, they have a number of carbon atoms for each polar group equal to or greater than 3, while all 4-alkoxy-7-chloroquinoline series compounds are predicted not to be hepatotoxic.

The Minnow toxicity predicts the median lethal concentration (LC_50_) for Flathead Minnows, and values below -0.3 are predicted as high acute toxicity [46]. Only 4-alkoxy/amino-7-chloroquinolines **3** and **14**, with R = Pent, are both predicted to have high acute toxicity with values of -0.33 and -0.67, respectively.

Analyzing Table **2**, 4-amino-7-chloroquinolines **2** and **3** showed the broadest antimicrobial activity, with activity against Gram-(+) and Gram-(−) bacteria, and *Candida albicans* fungus. More than that, 4-amino-7-chloroquinolines **1-3**, with R = alkyl, were the most active against *C. albicans*, with halos close to 30 mm. Considering the standard deviation, 4-amino-7-chloroquinoline **3** presented a ZOI (31.30 mm) very close to Fluconazole (32.42 mm). According to the World Health Organization (WHO), *C. albicans* has been ranked and categorized as a fungal pathogen in the critical group, which can cause generalized and invasive infections, and has demonstrated microbial resistance [56].

This same compound, 4-amino-7-chloroquinoline **3**, was active against Gram-(+) bacteria, *Bacillus cereus* and *Staphylococcus aureus*, with halos greater than 15 mm. Furthermore, 4-amino-7-chloroquinoline **2** was active against the Gram-(−) bacterium *Escherichia coli*, whose bacterial infection constitutes a neglected tropical disease [57].

On the other hand, the best results against *Pseudomonas aeruginosa* occurred for 4-amino-7-chloroquinolines **6-8**, when R = alkylamino, with halos greater than 20 mm. *P. aeruginosa*, capable of surviving even in environments with few nutrients, is an opportunistic pathogen, *i.e.*, it rarely causes disease in a healthy immune system, but takes advantage of immunocompromised and burned patients to establish an infection. It typically infects the respiratory, urinary and auditory systems, burns, and can cause bacteremia, a blood infection. Due to its ability to form biofilms and develop multiresistance to antimicrobial and antiseptic agents [58], this microorganism is one of the main causes of hospital infections with a high mortality rate [59].

Regarding Amoxicillin/Clavulanic acid and Tetracycline, 4-amino-7-chloroquinolines **6-8** were equal or more active than these antibiotic controls, while Ciprofloxacin antibiotic control presented a halo close to 30 mm. Although Ciprofloxacin, a fluoroquinolone, covers both Gram-(+) and Gram-(−) bacterial infections, recent studies have raised concerns that fluoroquinolone use may be associated with an increased risk of aortic aneurysm and dissection [60], among other side effects [61]. Therefore, it is essential to search for alternative antimicrobial agents to Ciprofloxacin.

In a previous work, compound **6** (10 mM) inhibited the growth of *Penicillium marneffei* by 50% [14]. In another study, compound **6 ** has been evaluated for its * in vitro* antibacterial activity against *E. coli* and *S. aureus*, and antifungal activity against *Aspergillus niger* and *C. albicans*, using Penicillin as an antibiotic control and Fluconazole as an antifungal control. Regarding the activity against strains of *C. albicans* (MTCC 871), *E. coli* (MTCC 119) and *S. aureus* (MTCC 96), compound **6** inhibited growth by 76.87, 78.5 and 85.28%, respectively [15].

Finally, the best results for the 4-alkoxy-7-chloroquinoline series were against *C. albicans*, when R = alkyl, with halos greater than 15 mm. These results were not as impressive as those of the 4-amino-7-chloroquinoline series, but linear alkyl side chains have been shown to be an indicative way of improving antimicrobial activity.

Analogous quinolines had their physicochemical, antifungal and toxicological properties studied. As a result, 2-methylquinoline (log P = 2.45) and 4-ethyl 2-methylquinoline (log P = 3.09) were active against *Candida* species, with Minimum Inhibitory Concentration (MIC) values ≥ 50 and 25-50 µg mL^-1^, respectively (Fluconazole, MIC 2-128 µg mL^-1^). Furthermore, 4-ethyl 2-methylquinoline, the most promising compound, did not show cytotoxic action [62]. These results corroborate the best results observed in the present work, *i.e.*, alkyl chain at 4-position.

Analyzing Table **4**, 4-amino-7-chloroquinolines **2** and **3**, and 4-alkoxy-7-chloroquinolines **13** and **14**, with R = Pr and Pent, respectively, are highly toxic. When lipophilicity decreases, toxicity on *A. salina* larvae also decreases, as for 4-amino-7-chloroquinoline **1,** with R = Me, which is moderately toxic.

**Fig. (1):**
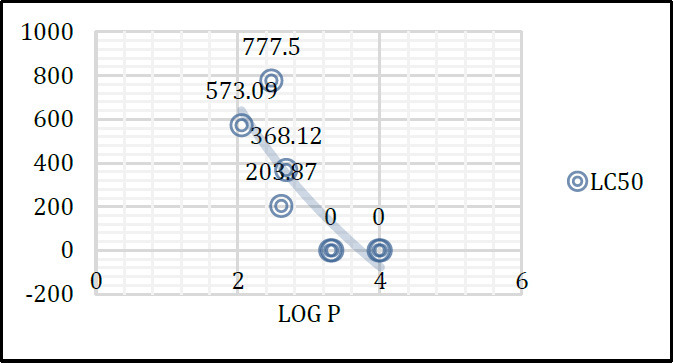
Lipophilicity-toxicity correlation trend for 4-amino-7-chloroquinolines (**1-3** and **6-8**) and 4-alkoxy-7-chloroquinolines (**13-14**). Log P: octanol/water partition coefficient prediction; LC_50_ (µg mL^-1^): lethal concentration value where a substance causes the death of 50% of *A. salina* larvae.

On the other hand, 4-amino-7-chloroquinolines **6** and **7** are slightly toxic. Coherently, for 4-amino-7-chloroquinoline **8**, which is moderately toxic, both toxicity on *A. salina* larvae and lipophilicity increased. It seems that increased lipophilicity in the side chain is harmful to *A. salina* larvae (Fig. **1**). These results are in agreement with Minnow toxicity prediction, where compounds **3** and **14**, with R = Pent, are both predicted to have high acute toxicity.

Considering the potential disposal of drugs in the aquatic environment, *in vitro* toxicity evaluation on *A. salina* larvae has been used as a possible indicator of ecotoxicity, and may discriminate cytotoxic compounds [63]. Furthermore, 4-ethyl 2-methylquinoline, analogous to the quinolines obtained in the present work, did not show cytotoxicity on Vero cells at concentrations related to its MIC values (25-50 µg mL^-1^), and a high percentage of viable cells was observed even at the highest concentration tested (100 µg mL^-1^) [62].

## 5 STUDY LIMITATIONS

Regarding the limitations of this study, 4-alkoxy/amino-7-chloroquinolines may have more selective molecular targets. However, toxicity studies need to be expanded to include cytotoxicity evaluation, especially for 4-amino-7-chloroquinolines 6 and 7, which are slightly toxic to A. salina larvae, with LC_50_ values of 573.09 and 777.50 µg mL^-1^, respectively. Furthermore, antimicrobial studies on different strains, using the broth microdilution method to determine MIC values, should be conducted to provide more robust data.

## CONCLUSION

With the aim of investigating the influence of the side chain on lipophilic, antimicrobial and toxicity properties and their correlations, a series of 19 compounds, analogues of chloroquine and hydroxychloroquine, was designed by a cleaner and facile approach, without the use of solvent and additional purification step, in high yields of 81-100%. Thus, 4-alkoxy/amino-7-chloroquinolines were evaluated for their potential as drugs through *in silico* ADMET test, which do not violate any Lipinski's Rule of Five as well as Veber parameters. The best antimicrobial results occurred for the 4-amino-7-chloroquinoline series against *Candida albicans*, when R = alkyl, with halos close to 30 mm, and against *Pseudomonas aeruginosa*, when R = alkylamino, with halos greater than 20 mm. The most promising antimicrobial agents were evaluated for their *in vitro* toxicity on *Artemia salina* larvae. At the same time, we can observe a correspondence between Minnow toxicity prediction and *in vitro* toxicity on *A. salina* larvae, where compounds **3** and **14**, with R = Pent, were both predicted to have high acute toxicity (log LC_50_ < -0.3) and classified as highly toxic (LC_50_ < 100 µg mL^-1^); linear alkyl sides appear to be an assertive way to improve antimicrobial activity. Now, we are motivated to continue antimicrobial studies for compounds **1-3** and **6-8** with greater activity against *C. albicans* and *P. aeruginosa*, respectively, by the determination of MIC values, especially for 4-amino-7-chloroquinolines **6** and **7**, which are slightly toxic on *A. salina* larvae (LC_50_ 500-1000 µg mL^-1^).

## Figures and Tables

**Table 1:** ADMET prediction for 4-alkoxy/amino-7-chloroquinolines (1-19).

**Compound**, **R**	**Lipinski**				**Veber**		**Toxicity**		
	**MW** (Da)	**HBD**	**HBA**	**Log P**	**TPSA** (Å^2^)	**Nrotb**	**Skin Sensitisation**	**Hepato**	**Minnow** log LC_50_
**1**, Me	192.64	1	1	2.61	24.92	1	No	No	0.67
**2**, Pr	220.70	1	1	3.30	24.92	3	No	No	0.19
**3**, Pent	248.75	1	1	3.98	24.92	5	No	Yes	-0.33
**4**, (CH_2_)_2_NMe_2_	249.74	1	2	2.69	28.16	4	No	No	1.74
**5**, (CH_2_)_2_NEt_2_	277.79	1	2	3.35	28.16	6	No	Yes	1.41
**6**, (CH_2_)_2_NH_2_	221.69	2	2	2.05	50.94	3	No	No	0.96
**7**, (CH_2_)_3_NH_2_	235.71	2	2	2.47	50.94	4	No	Yes	0.66
**8**, (CH_2_)_4_NH_2_	249.74	2	2	2.68	50.94	5	No	Yes	0.37
**9**, (CH_2_)_6_NH_2_	277.79	2	2	3.32	50.94	7	No	Yes	-0.08
**10**, (CH_2_)_2_OH	222.67	2	2	2.14	45.15	3	No	No	0.97
**11**, (CH_2_)_3_OH	236.70	2	2	2.56	45.15	4	No	Yes	0.67
**12**, (CH_2_)_2_NH(CH_2_)_2_OH	265.74	3	3	1.98	57.18	6	No	No	2.17
**13**, Pr	221.68	0	2	3.33	22.12	3	No	No	-0.14
**14**, Pent	249.74	0	2	4.01	22.12	5	No	No	-0.67
**15**, (CH_2_)_2_OH	223.66	1	3	2.18	42.35	3	No	No	0.64
**16**, (CH_2_)_3_OH	237.68	1	3	2.50	42.35	4	No	No	0.34
**17**, (CH_2_)_4_OH	251.71	1	3	2.80	42.35	5	No	No	0.05
**18**, (CH_2_)_2_O(CH_2_)_2_OH	267.71	1	4	2.20	51.58	6	No	No	-0.08
**19**, (CH_2_)_2_S(CH_2_)_2_OH	283.77	1	3	2.84	67.65	6	No	No	-0.11

**Abbreviations:** MW: molecular weight; HBD: hydrogen bond donor; HBA: hydrogen bond acceptor; Log P: octanol/water partition coefficient; TPSA: topological polar surface area; nrotb: number of rotatable bonds; Minnow Toxicity: median lethal concentration (LC_50_) for Flathead Minnows.

**Note:** Lipinski: MW < 500 Da, HBD ≤ 5, HBA ≤ 10, log *p* ≤ 5 [22]; and Veber parameters: TPSA ≤ 140 Å^2^, nrotb ≤ 10 [23].

**Table 2:** ZOI (mm) ± SD for 50 µL of 4-alkoxy/amino-7-chloroquinolines (1-19).

**Compound**, R	**Bacteria**					**Yeast fungus**
	**Gram-(+)**			**Gram-(−)**		
	***B. cereus* CCT 0198**	***E. faecalis* NEWP 0012**	***S. aureus* ATCC 25923**	***E. coli* NEWP 0039**	***P. aeruginosa* NEWP 0027**	***C. albicans* NEWP 0031**
**1**, Me	-	8.61 ± 0.69	7.58 ± 0.17	13.39 ± 0.00	6.35 ± 0.00	24.63 ± 0.24
**2**, Pr	11.69 ± 0.61	9.87 ± 0.18	10.97 ± 1.06	14.52 ± 0.50	7.12 ± 0.08	20.10 ± 0.98
**3**, Pent	17.96 ± 0.31	13.79 ± 0.51	15.40 ± 0.10	12.39 ± 0.38	6.35 ± 0.00	29.59 ± 1.71
**4**, (CH_2_)_2_NMe_2_	7.67 ± 0.31	-	7.50 ± 0.31	8.32 ± 0.81	6.95 ± 0.36	12.37 ± 0.28
**5**, (CH_2_)_2_NEt_2_	7.92 ± 1.47	-	7.39 ± 0.45	8.57 ± 0.04	6.75 ± 0.17	8.51 ± 0.63
**6**, (CH_2_)_2_NH_2_	10.32 ± 0.42	-	7.83 ± 0.75	9.61 ± 0.83	21.88 ± 1.31	8.60 ± 0.18
**7**, (CH_2_)_3_NH_2_	8.93 ± 0.10	-	7.81 ± 0.52	8.54 ± 0.46	20.91 ± 0.65	7.60 ± 0.26
**8**, (CH_2_)_4_NH_2_	7.73 ± 0.39	-	6.59 ± 0.21	7.81 ± 0.18	21.90 ± 0.97	6.28 ± 0.07
**9**, (CH_2_)_6_NH_2_	8.83 ± 1.20	-	8.27 ± 0.25	7.87 ± 0.18	7.37 ± 0.18	6.72 ± 0.05
**10**, (CH_2_)_2_OH	-	-	-	6.68 ± 0.12	-	7.14 ± 0.40
**11**, (CH_2_)_3_OH	-	-	6.44 ± 0.12	6.61 ± 0.47	7.05 ± 0.62	8.31 ± 0.48
**12**, (CH_2_)_2_NH(CH_2_)_2_OH	-	-	7.55 ± 0.35	6.82 ± 0.12	-	7.78 ± 0.19
**13**, Pr	9.51 ± 0.45	9.08 ± 0.38	13.73 ± 2.06	7.40 ± 0.11	-	15.90 ± 0.77
**14**, Pent	8.35 ± 0.04	9.30 ± 0.85	9.64 ± 0.34	-	-	14.83 ± 1.35
**15**, (CH_2_)_2_OH	-	-	7.19 ± 0.57	6.07 ± 0.04	6.70 ± 0.42	6.91 ± 0.17
**16**, (CH_2_)_3_OH	6.62 ± 0.10	8.58 ± 0.43	6.43 ± 0.04	-	-	11.71 ± 1.42
**17**, (CH_2_)_4_OH	7.68 ± 0.58	-	8.22 ± 0.40	6.19 ± 0.04	6.93 ± 0.04	12.34 ± 2.35
**18**, (CH_2_)_2_O(CH_2_)_2_OH	6.92 ± 0.21	-	7.71 ± 0.22	6.75 ± 0.20	6.71 ± 0.20	7.44 ± 0.06
**19**, (CH_2_)_2_S(CH_2_)_2_OH	8.64 ± 0.29	-	6.74 ± 0.29	6.11 ± 0.07	6.04 ± 0.01	13.23 ± 1.06
AMC (30 µg)	-	-	41.43 ± 2.71	15.30 ± 3.72	19.90 ± 2.14	NA
CIP (5 µg)	23.20 ± 2.11	16.79 ± 2.17	33.49 ± 1.58	25.99 ± 1.22	29.44 ± 2.22	NA
PEN (10 U.I.)	-	16.96 ± 0.90	45.11 ± 4.30	-	-	NA
TET (30 µg)	26.22 ± 2.41	14.97 ± 0.31	30.04 ± 0.99	21.06 ± 0.39	21.06 ± 0.39	NA
VAN (30 µg)	12.15 ± 4.23	16.65 ± 0.36	19.79 ± 1.71	-	-	NA
FCZ (32 µg)	NA	NA	NA	NA	NA	32.42 ± 0.00

**Abbreviations:** ZOI: Zone of Inhibition; SD: Standard Deviation; AMC: Amoxicillin/Clavulanic acid; CIP: Ciprofloxacin; PEN: Penicillin G; TET: Tetracycline; VAN: Vancomycin; FCZ: Fluconazole; NA: Not Applicable; -: Not Active.

**Table 3:** AP ± SD (%) of alive *A. salina* nauplii in different concentrations of 4-amino-7-chloroquinolines (1-3 and 6-8) and 4-alkoxy-7-chloroquinolines (13-14).

**Compound**, R	**Concentration Series** (µg mL^-1^)		
	**125**	**250**	**375**
**1**, Me	68.94 ± 5.97	44.82 ± 5.97	13.79 ± 5.97
**2**, Pr	6.89 ± 5.97	3.44 ± 5.97	0.00 ± 0.00
**3**, Pent	3.44 ± 5.97	0.00 ± 0.00	0.00 ± 0.00
**6**, (CH_2_)_2_NH_2_	75.84 ± 5.97	65.50 ± 5.97	62.05 ± 0.00
**7**, (CH_2_)_3_NH_2_	95.38 ± 4.00	82.73 ± 0.0	68.94 ± 5.97
**8**, (CH_2_)_4_NH_2_	91.93 ± 8.69	68.94 ± 5.97	51.71 ± 0.00
**13**, Pr	6.89 ± 5.97	3.44 ± 5.97	0.00 ± 0.00
**14**, Pent	6.89 ± 5.97	0.00 ± 0.00	0.00 ± 0.00

**Abbreviations:** AP: Average Percentage; SD: Standard Deviation.

**Note:** Statistics were analyzed using the ANOVA method in a confidence interval with a confidence level of 95%.

**Table 4:** LC_50_ values of 4-amino-7-chloroquinolines (1-3 and 6-8) and 4-alkoxy-7-chloroquinolines (13-14) on *A. salina* larvae.

**Structure**	**Compound, R**	**Log P**	**LC_50_ (µg mL^-1^)**
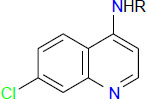	**1**, Me	2.61	203.87
	**2**, Pr	3.30	<< 100
	**3**, Pent	3.98	<< 100
	**6**, (CH_2_)_2_NH_2_	2.05	573.09
	**7**, (CH_2_)_3_NH_2_	2.47	777.50
	**8**, (CH_2_)_4_NH_2_	2.68	368.12
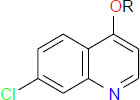	**13**, Pr	3.33	<< 100
	**14**, Pent	4.01	<< 100

**Abbreviations:** Log P: octanol/water partition coefficient prediction; LC_50_: lethal concentration value where a substance causes the death of 50% of *A. salina* larvae.

**Table 5:** Some reactional conditions described in the literature to obtain 4-alkoxy/amino-7-chloroquinolines *via* S_N_Ar reaction between 4,7-dichloroquinoline and alcohols/amines.

**Compound**, R	**Additional**			**T (°C)**	**t (h)**	**Separation Step**	**Yield (%)**	**Reference**
	**Reagent**	**Solvent**	**Condition**					
**1**, Me	-	DMSO	-	70	216	Precipitation in water	98	[30]
**2**, Pr	-	-	-	Reflux	12	Precipitation in water	80	[31]
**3**, Pent	-	EtOH	-	Reflux	18	Column chromatography	-	[32]
**4**, (CH_2_)_2_NMe_2_	-	-	Sealed tube	110	16	Column chromatography	58	[33]
**5**, (CH_2_)_2_NEt_2_	-	Phenol	-	90	11	Column chromatography	35	[34]
**6**, (CH_2_)_2_NH_2_	-	-	US	-	0,5	Precipitation in water	97	[35]
**7**, (CH_2_)_3_NH_2_	-	-	-	80-135	4	Column chromatography	90	[36]
**8**, (CH_2_)_4_NH_2_	-	-	-	80-135	4	Column chromatography	75	[36]
**9**, (CH_2_)_6_NH_2_	-	-	-	80-135	4	Column chromatography	55	[36]
**10**, (CH_2_)_2_OH	-	EtOH	-	Reflux	8	Precipitation in water	84	[37]
**11**, (CH_2_)_3_OH	-	EtOH	-	Reflux	8	Precipitation in water	82	[37]
**12**, (CH_2_)_2_NH(CH_2_)_2_OH	NaI	Phenol	-	Reflux	-	Distillation with steam	74	[38]
**13**, Pr	HCl (0,5 *N*)	-	-	Reflux	24	Precipitation in water	66	[39]
**14**, Pent	*t*-BuOK	-	-	100	8	Precipitation in water	88	In this work.
**15**, (CH_2_)_2_OH	*t*-BuOK	*t*-BuOH	N_2(g)_	80	16	Liquid-liquid extraction	94	[37]
**16**, (CH_2_)_3_OH	*t*-BuOK	*t*-BuOH	N_2(g)_	80	16	Liquid-liquid extraction	96	[37]
**17**, (CH_2_)_4_OH	*t*-BuOK	*t*-BuOH	N_2(g)_	80	18	Liquid-liquid extraction	66	[9]
**18**, (CH_2_)_2_O(CH_2_)_2_OH	*t*-BuOK	-	-	100	8	Precipitation in water	82	In this work.
**19**, (CH_2_)_2_S(CH_2_)_2_OH	*t*-BuOK	-	-	100	8	Precipitation in water	85	In this work.

**Abbreviations:** -: Not Informed; US: Ultrasound Irradiation.

## Data Availability

The data and supportive information are available within the article.

## LIST OF ABBREVIATIONS

ADME: Absorption, Distribution, Metabolism and Excretion
HRMS: High-Resolution Mass Spectrometry
ESI: Electrospray Ion
TLC: Thin Layer Chromatography
CLSI: Clinical and Laboratory Standards Institute
