# Supplementary material for: Side Chain Effects on the Lipophilicity-antimicrobial-toxicity Correlation of Greener 4-Alkoxy/Amino-7-Chloroquinolines
Source: Curr Med Chem. 2025 Oct 8;33(5):924–38. doi: 10.2174/0109298673372039250614231629 (PMC13223418; doi:10.2174/0109298673372039250614231629)
Supplement: Supplementary file 1 [file CMC-33-5-924_SD1.pdf]

Supplementary Material

Side Chain Effects on the Lipophilicity-antimicrobial-toxicity Correlation of Greener 4-Alkoxy/Amino-7-Chloroquinolines

Gabriela F. Fiss[1,*], Everton P. Silva[1], Maria F. S. Madruga[1], Abraão P. Sousa[1], Helivaldo D.S. Souza[1], Rádamis B. Castor[2], Maria H. Nascimento[2], Krystyna G. Lira[2] and Petrônio F. Athayde-Filho[1]

[1]LPBS, Department of Chemistry, Federal University of Paraíba, João Pessoa, Brazil; [2]Department of Molecular Biology, Federal University of Paraíba, João Pessoa, Brazil

Full NMR, IR and HRMS spectra of 4-alkoxy-7-chloroquinolines (14, 18 and 19)

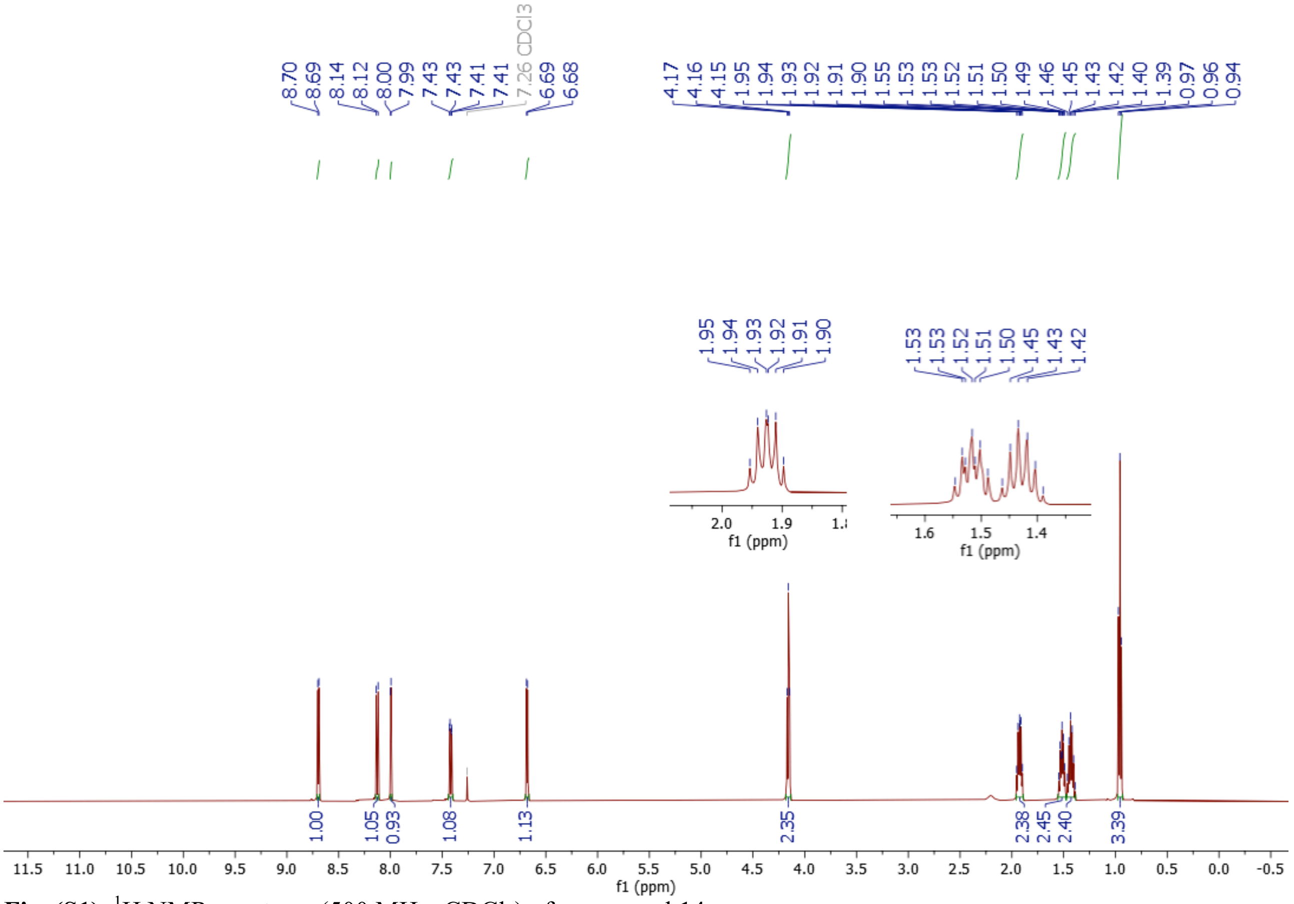

Fig. (S1). ^{1}H NMR spectrum (500 MHz, $CDCl_3$) of compound **14**.

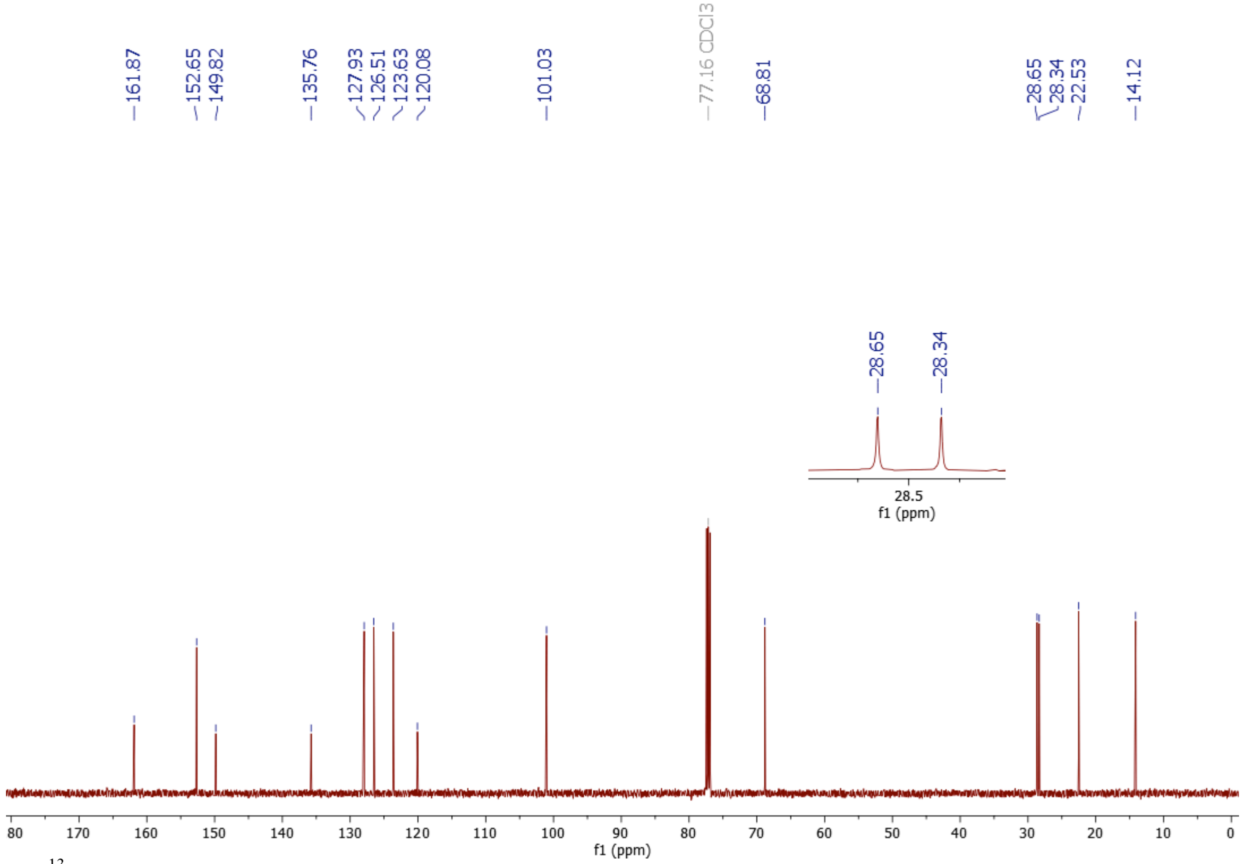

Fig. (S2). ^{13}C NMR spectrum (126 MHz, $CDCl_3$) of compound **14**.

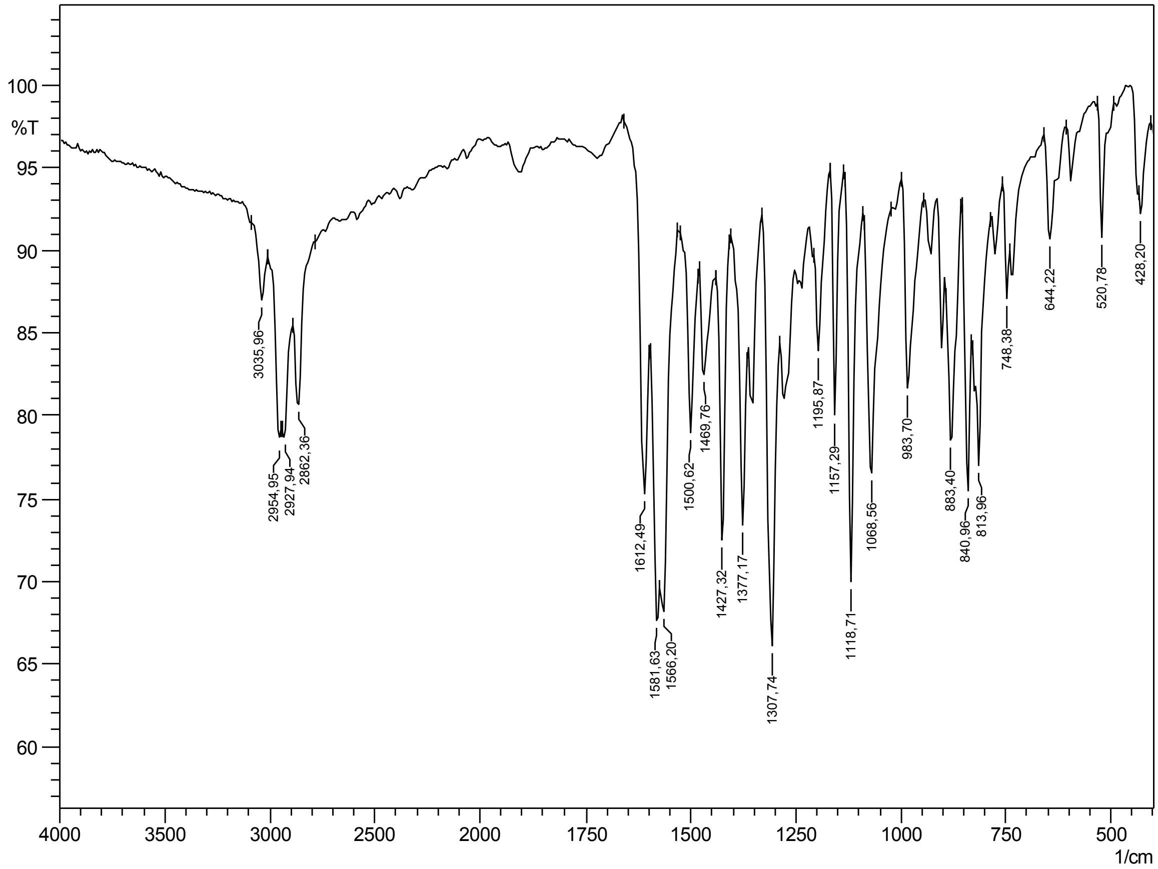

Fig. (S3). IR spectrum (KBr) of compound **14**.

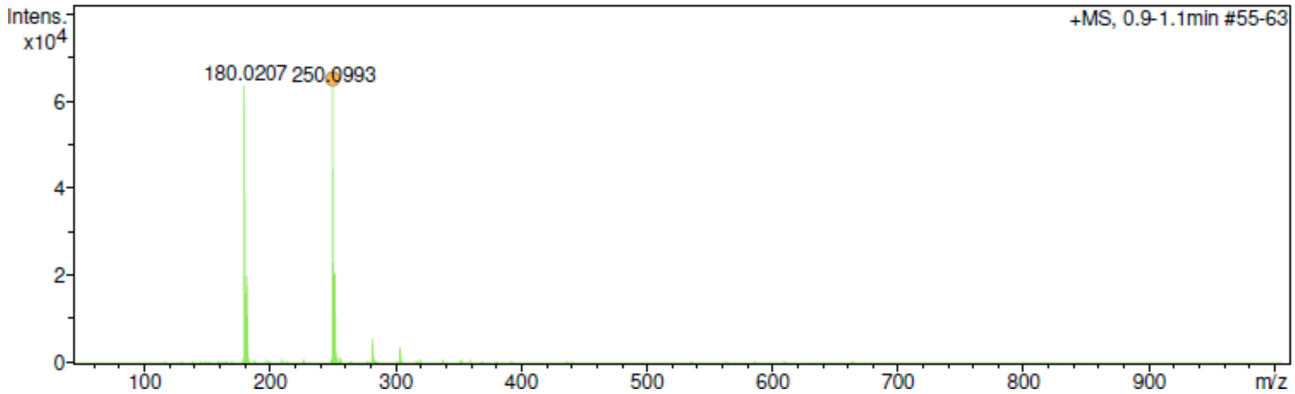

Fig. (S4). HRMS spectrum (ESI) of compound **14**.

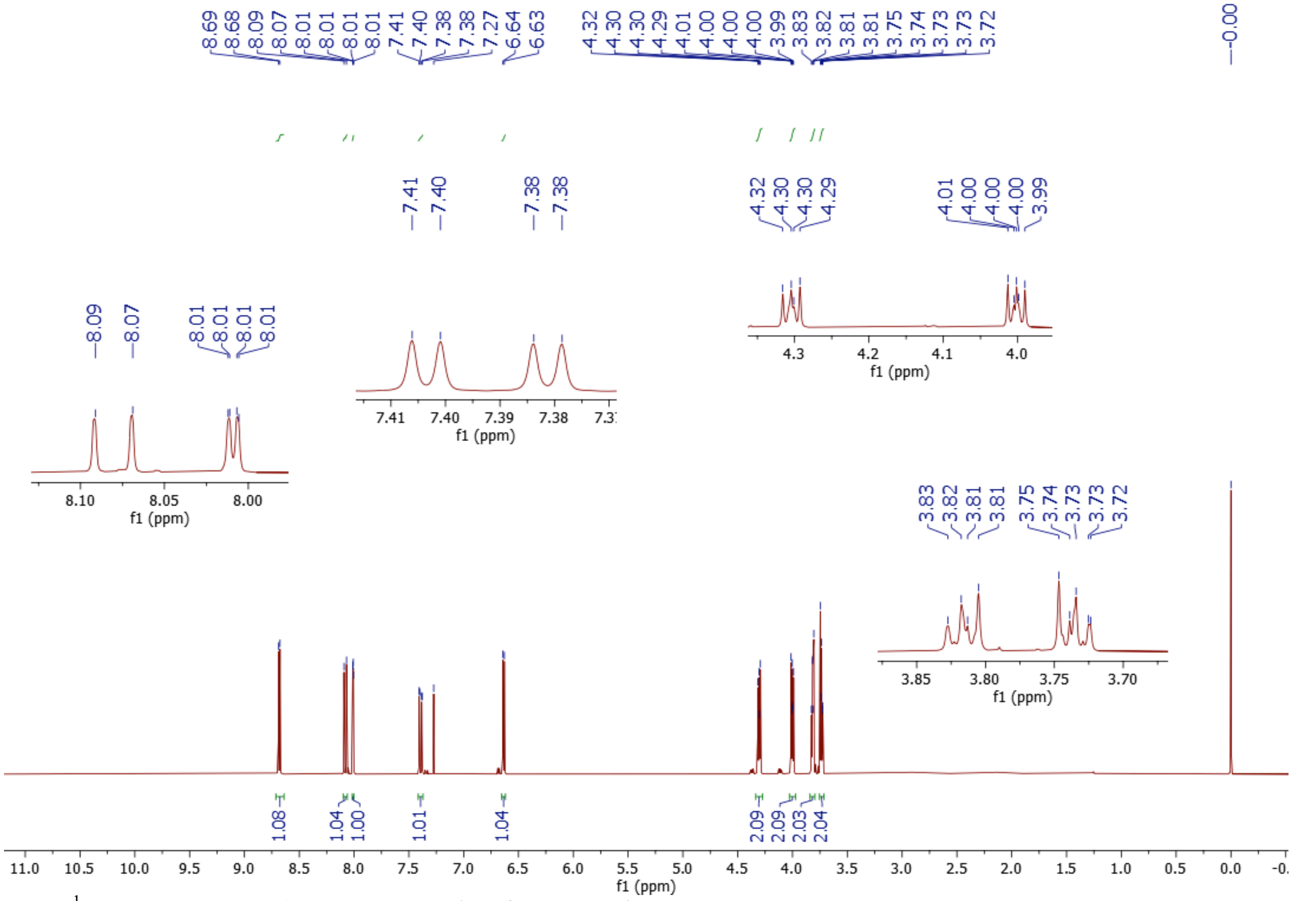

Fig. (S5). ^{1}H NMR spectrum (400 MHz, $CDCl_3$) of compound **18**.

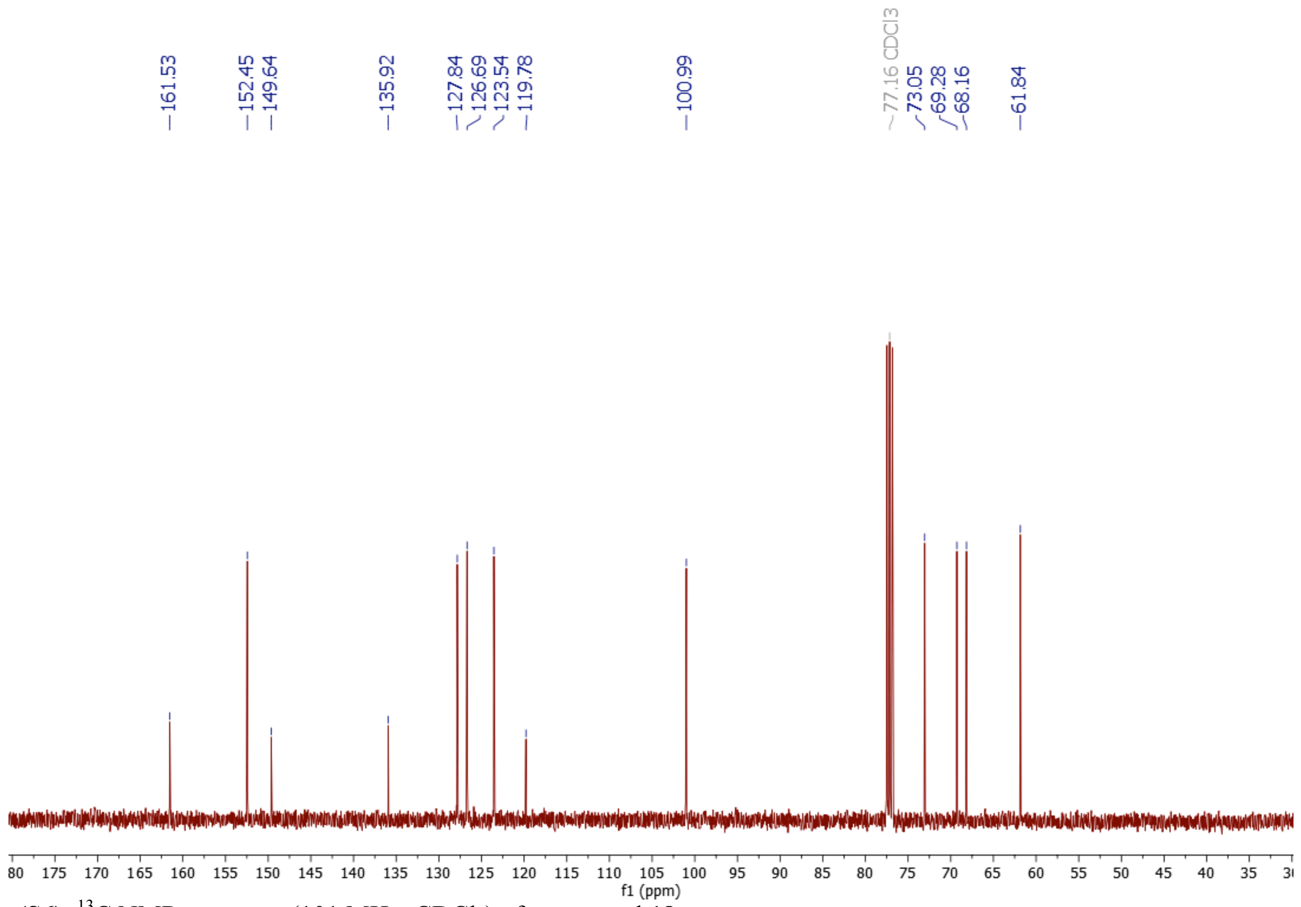

Fig. (S6). ^{13}C NMR spectrum (101 MHz, $CDCl_3$) of compound **18**.

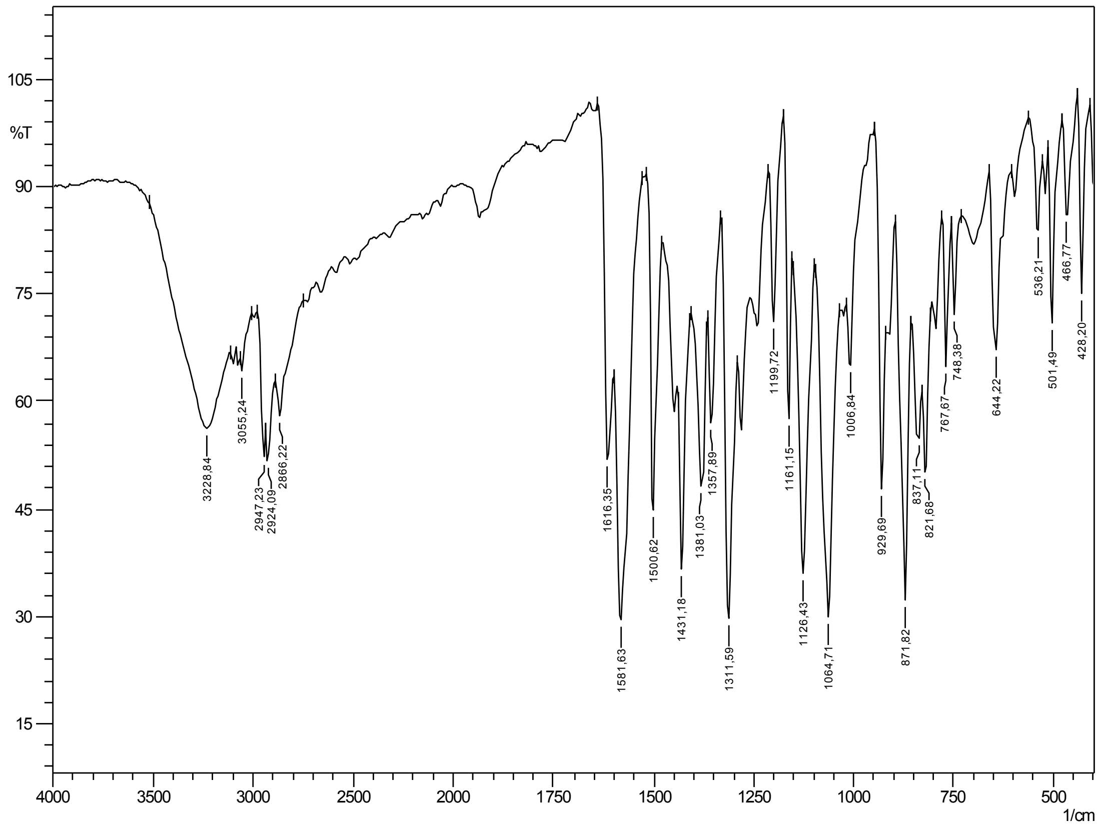

Fig. (S7). IR spectrum (KBr) of compound **18**.

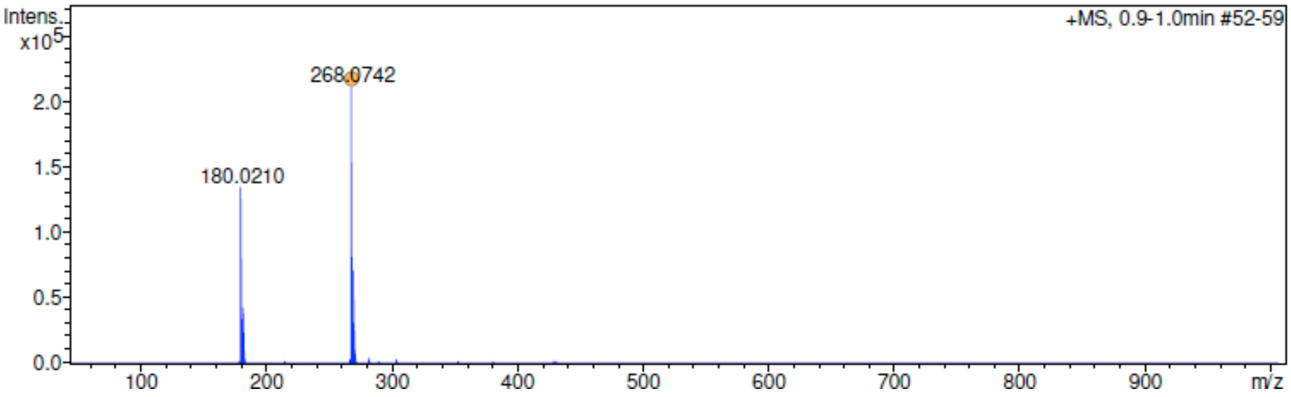

Fig. (S8). HRMS spectrum (ESI) of compound **18**.

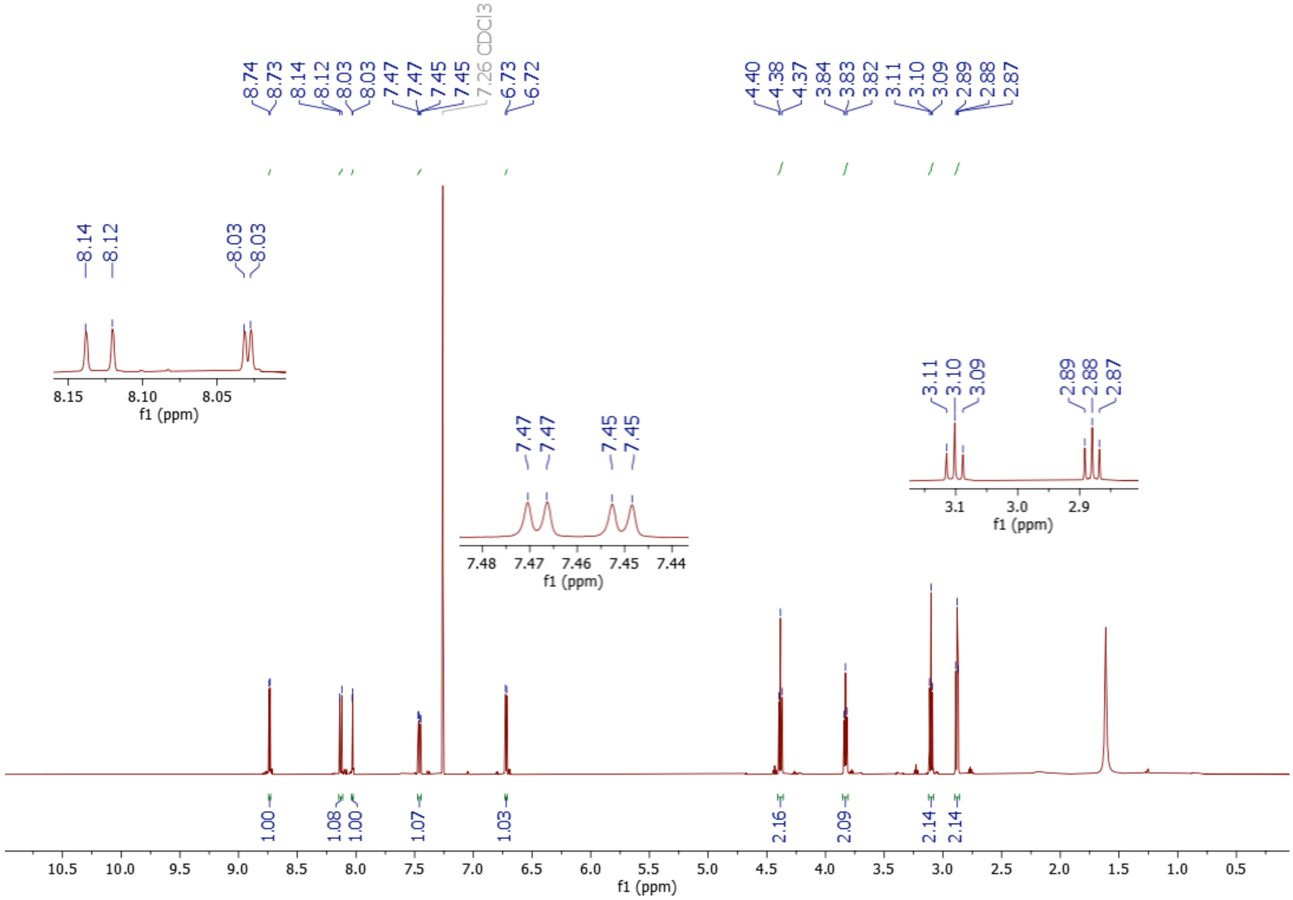

Fig. (S9). ^{1}H NMR spectrum (500 MHz, $CDCl_3$) of compound **19**.

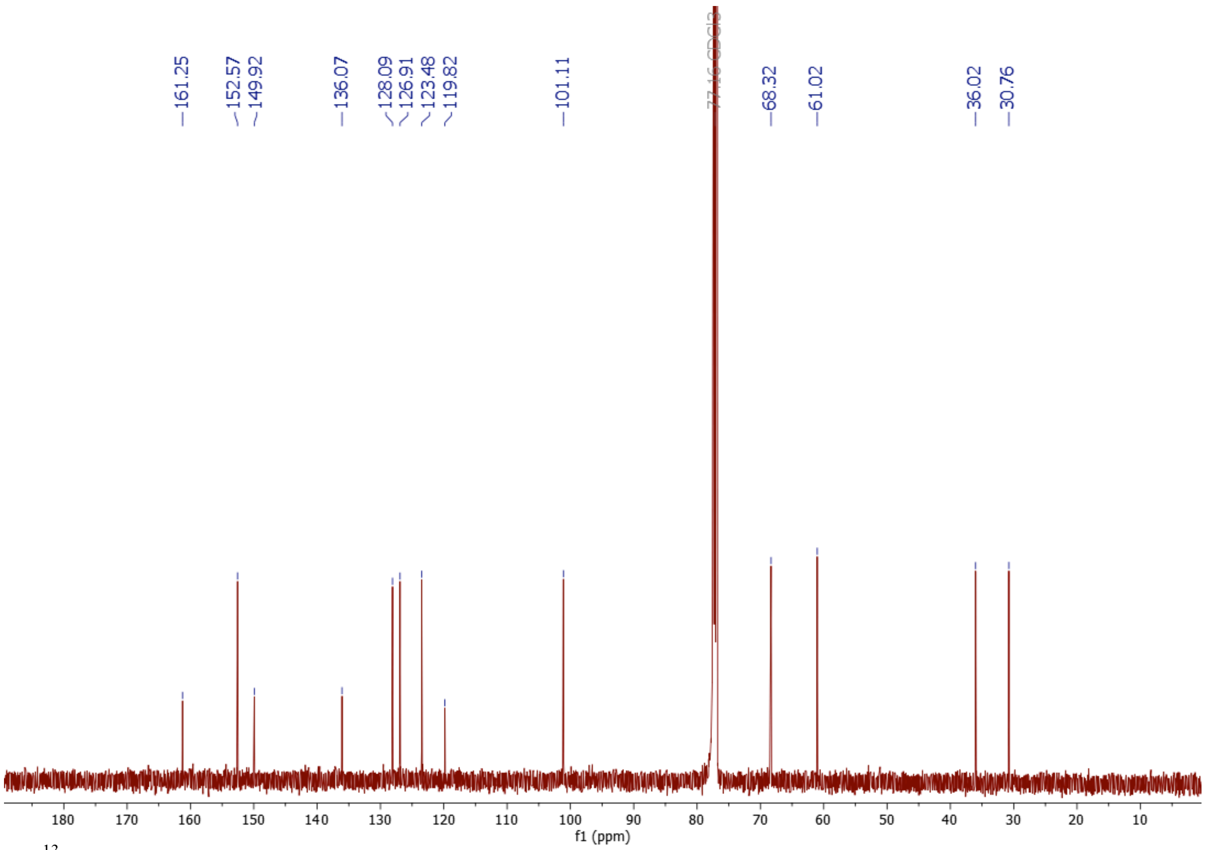

Fig. (S10). ^{13}C NMR spectrum (126 MHz, $CDCl_3$) of compound **19**.

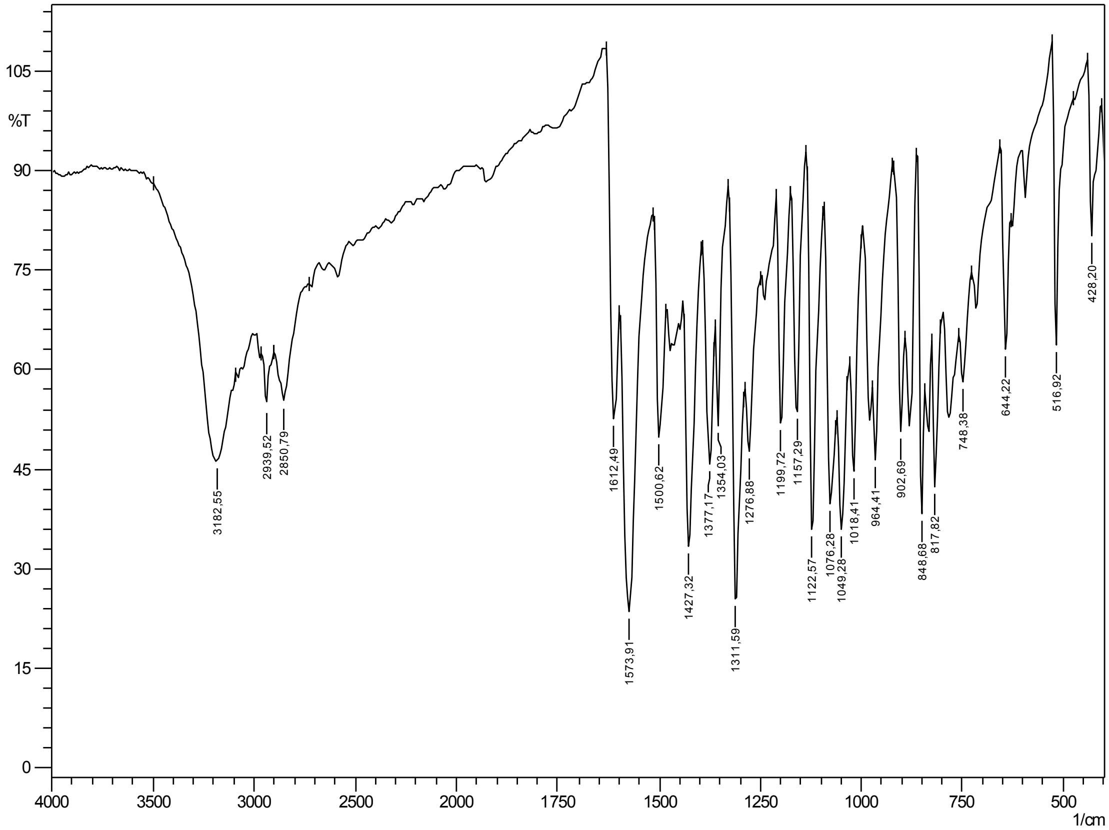

Fig. (S11). IR spectrum (KBr) of compound **19**.

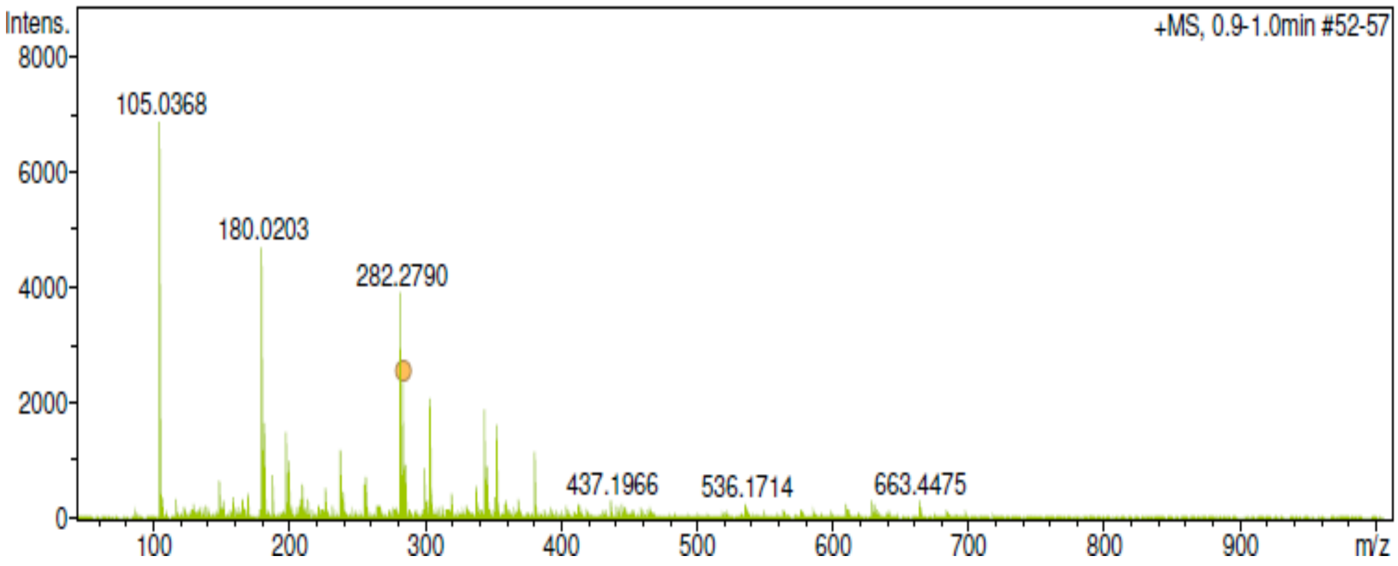

Fig. (S12). HRMS spectrum (ESI) of compound **19**.

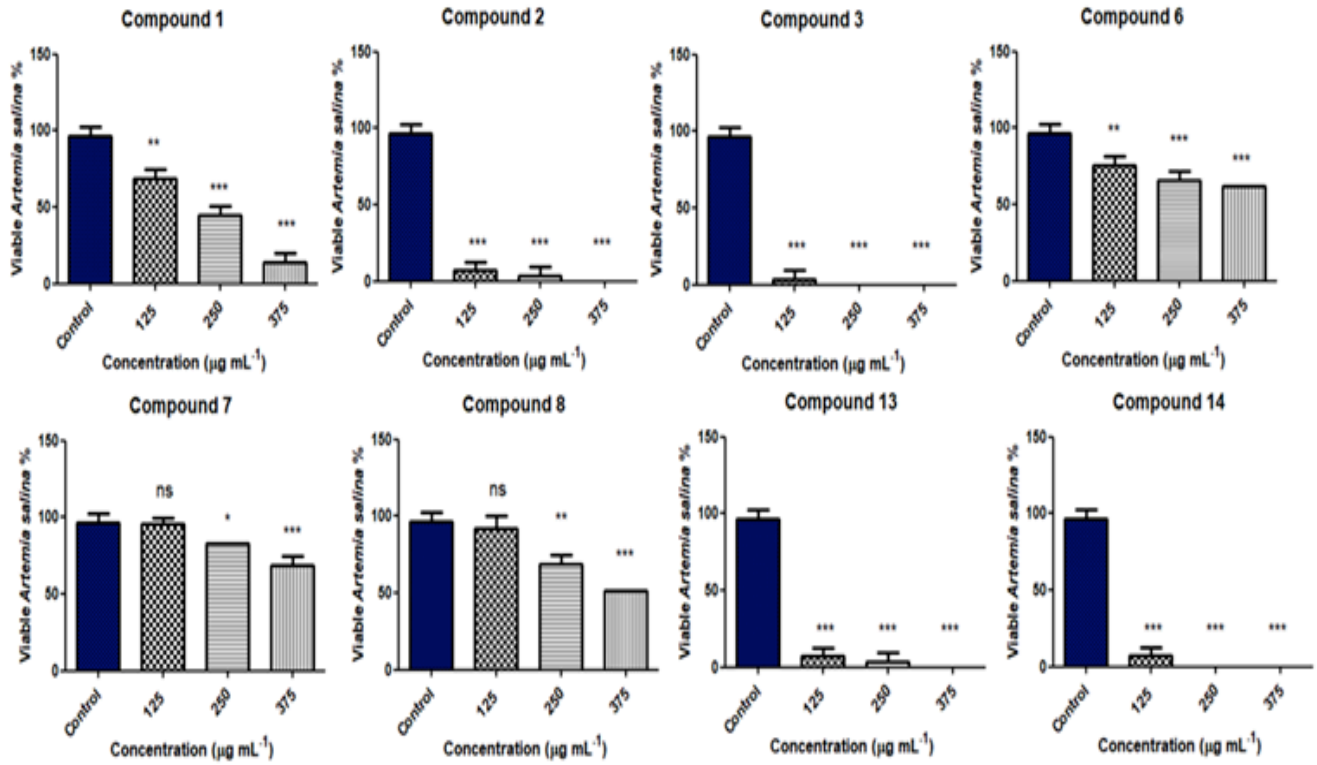

Fig. (S13). Average percentages of alive *A. salina* nauplii in different concentrations of 4-amino-7-chloroquinolines (**1-3** and **6-8**) and 4-alkoxy-7-chloroquinolines (**13-14**). Means were significantly different when P ≤ 0.0002***. ns: non-significant.

Table S1. Estimated median lethal concentration (LC_{50}) for 4-amino-7-chloroquinolines (1-3 and 6-8) and 4-alkoxy-7-chloroquinolines (13-14) on *Artemia salina* larvae using linear regression equation.

Compound, R	Graphical
**1**, Me	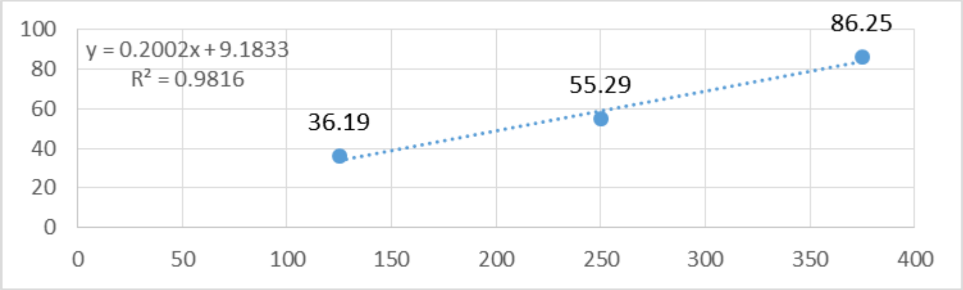
**2**, Pr	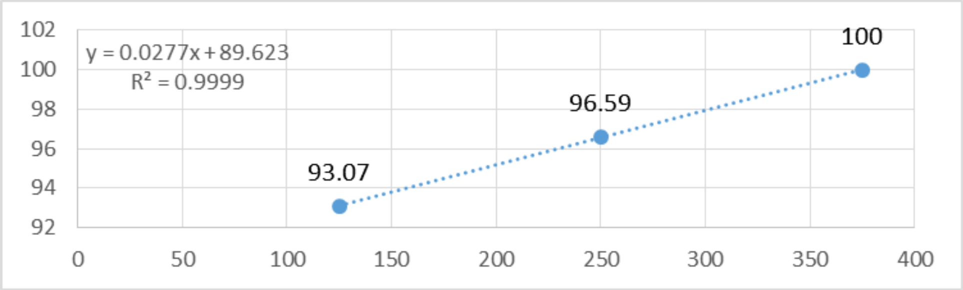
**3**, Pent	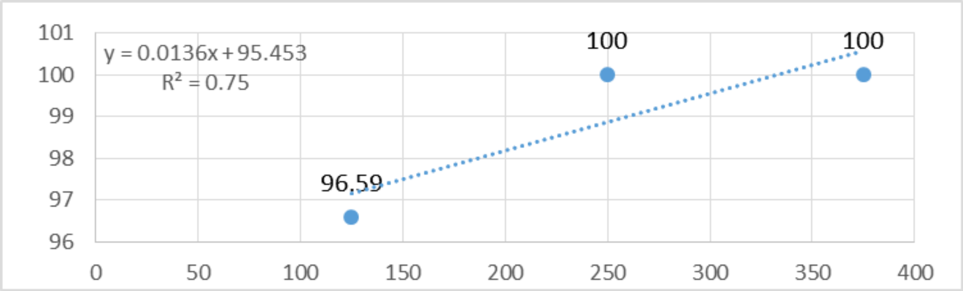
**6**, $(CH_2)_2NH_2$	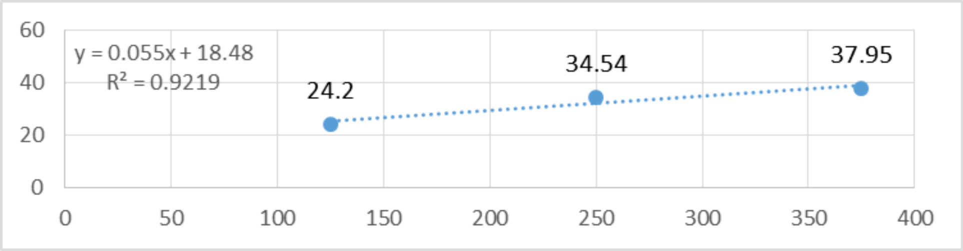
**7**, $(CH_2)_3NH_2$	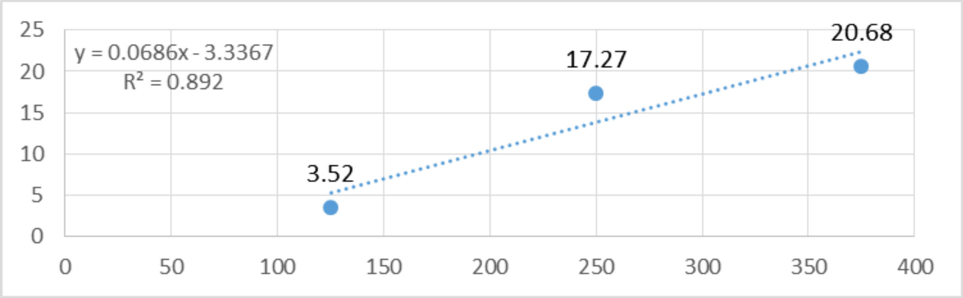

**8**, $(CH_2)_4NH_2$	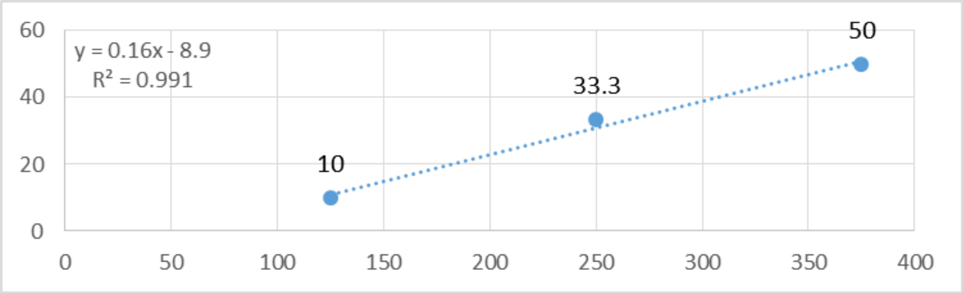
**13**, Pr	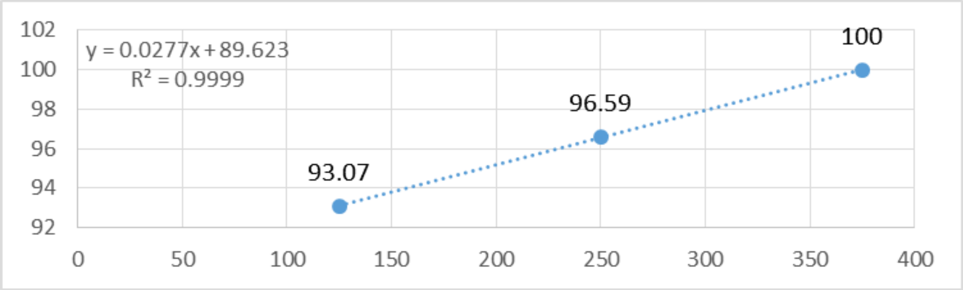
**14**, Pent	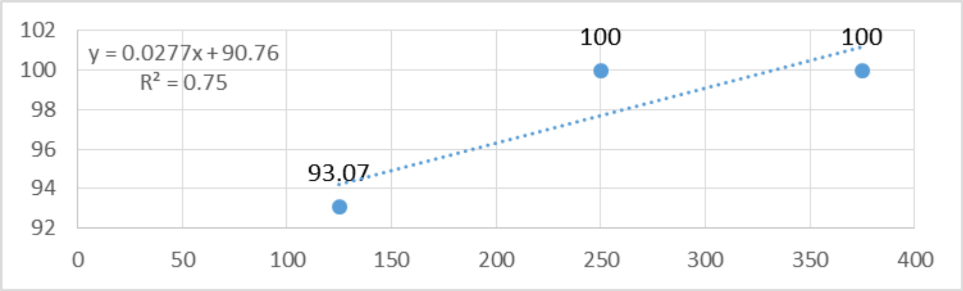
